# EF-hand calcium sensor, EfhP, controls transcriptional regulation of iron uptake by calcium in *Pseudomonas aeruginosa*

**DOI:** 10.1128/mbio.02447-24

**Published:** 2024-10-22

**Authors:** Jacob Burch-Konda, Biraj B. Kayastha, Myriam Achour, Aya Kubo, Mackenzie Hull, Reygan Braga, Lorelei Winton, Rendi R. Rogers, Erika I. Lutter, Marianna A. Patrauchan

**Affiliations:** 1 Department of Microbiology and Molecular Genetics, Oklahoma State University, Stillwater, Oklahoma, USA; Emory University School of Medicine, Atlanta, Georgia, USA

**Keywords:** transcription, calcium signaling, promoter activity, pyoverdine, RNA seq, virulence, human pathogen, CarRS, BqsRS, Fur, CF clinical isolates

## Abstract

The human pathogen *Pseudomonas aeruginosa* (*Pa*) poses a major risk for a range of severe infections, particularly lung infections in patients suffering from cystic fibrosis (CF). As previously reported, the virulent behavior of this pathogen is enhanced by elevated levels of Ca^2+^ that are commonly present in CF nasal and lung fluids. In addition, a Ca^2+^-binding EF-hand protein, EfhP (PA4107), was partially characterized and shown to be critical for the Ca^2+^-regulated virulence in *P. aeruginosa*. Here, we describe the rapid (10 min, 60 min), and adaptive (12 h) transcriptional responses of PAO1 to elevated Ca^2+^ detected by genome-wide RNA sequencing and show that *efhP* deletion significantly hindered both rapid and adaptive Ca^2+^ regulation. The most differentially regulated genes included multiple Fe sequestering mechanisms, a large number of extracytoplasmic function sigma factors (ECFσ), and several virulence factors, such as the production of pyocins. The Ca^2+^ regulation of Fe uptake was also observed in CF clinical isolates and appeared to involve the global regulator Fur. In addition, we showed that the *efhP* transcription is controlled by Ca^2+^ and Fe, and this regulation required a Ca^2+^-dependent two-component regulatory system CarSR. Furthermore, the *efhP* expression is significantly increased in CF clinical isolates and upon pathogen internalization into epithelial cells. Overall, the results established for the first time that Ca^2+^ controls Fe sequestering mechanisms in *P. aeruginosa* and that EfhP plays a key role in the regulatory interconnectedness between Ca^2+^ and Fe signaling pathways, the two distinct and important signaling pathways that guide the pathogen’s adaptation to the host.

**IMPORTANCE:**

*Pseudomonas aeruginosa* (*Pa*) poses a major risk for severe infections, particularly in patients suffering from cystic fibrosis (CF). For the first time, kinetic RNA sequencing analysis identified *Pa* rapid and adaptive transcriptional responses to Ca^2+^ levels consistent with those present in CF respiratory fluids. The most highly upregulated processes include iron sequestering, iron starvation sigma factors, and self-lysis factors pyocins. An EF-hand Ca^2+^ sensor, EfhP, is required for at least 1/3 of the Ca^2+^ response, including the majority of the iron uptake mechanisms and the production of pyocins. Transcription of *efhP* itself is regulated by Ca^2+^ and Fe, and increases during interactions with host epithelial cells, suggesting the protein’s important role in *Pa* infections. The findings establish the regulatory interconnectedness between Ca^2+^ and iron signaling pathways that shape *Pa* transcriptional responses. Therefore, understanding Pa’s transcriptional response to Ca^2+^ and associated regulatory mechanisms will serve in the development of future therapeutics targeting *Pa*’s dangerous infections.

## INTRODUCTION

*Pseudomonas aeruginosa (Pa*) is a major human pathogen causing severe chronic infections. It is a primary pathogen infecting the airways of cystic fibrosis (CF) patients, leading to cellular damage and fatal obstructive lung disease (1, 2). *Pa* is also responsible for life-threatening infective endocarditis and infections in the urinary tract, skin, burns, and surgical wounds (3–6). *Pa* infections have a mortality rate of 18% to 60% and present a tremendous healthcare burden in the United States (7) and worldwide. *Pa* belongs to the ESKAPE group of pathogens with increased virulence, persistence, and transmissibility (8) and has been recognized by both the Centers for Disease Control and Prevention and the World Health Organization as a serious threat that requires the urgent development of new therapeutics. The success of *Pa* as a pathogen and the failure of antibiotic therapies to eliminate *Pa* infections are thought to be due in part to its physiological plasticity, enabling the adaptability to the host environment and resistance to both immune protection and antimicrobial therapies (reviewed in references 9–11). Among *Pa*’s key adaptive mechanisms are phenotypic diversification (12), acquisition of essential iron (13), stress responses (14, 15), persistence (16), and biofilm formation (17). Furthermore, *Pa* was shown to shape the host’s innate immune response to favor pathogen survival (18, 19). However, the host stimuli that trigger adaptive responses and the involved signaling cascades are not fully understood.

Upon entry into the host, pathogenic bacteria must survive several lines of the host’s innate and adaptive immune defenses (20), which requires efficient recognition and responsiveness to an array of host messengers, for example, iron (21, 22), potassium (23), interferon-γ (24), and defense peptides (25). To coordinate the responses to host factors, *Pa* possesses a record number of signaling and transcriptional regulatory systems, exemplified by cAMP/Vfr, quorum sensing, Gac/Rsm, c-di-GMP, MucA/HptB signaling, that modulate and tune the gene expression of multiple pathways required for survival, among others including production of extracellular polysaccharides, efflux systems, secretion systems, toxins, and iron uptake (26–28). Among a multitude of host factors that control the expression of *Pa* virulence factors, calcium (Ca^2+^) is particularly influential as it also plays a major role in a human host by controlling life-sustaining processes, such as cell division, differentiation, apoptosis, gene transcription, and immune responses (29–31). Disruption of Ca^2+^ homeostasis may cause or result from diseases, including bacterial infections and genetic disorders (32–40). One example is CF, where the levels of Ca^2+^ are elevated in the lungs and nasal fluids due to an imbalance of ion homeostasis (41–45). Cases of calcification of the lungs, vascular, and extravascular regions have also been reported (45–47). Such elevated levels of Ca^2+^ may act as a signaling cue and lead to modulation of the physiology and virulence of the invading bacteria, including *Pa (*40, 48–52). Therefore, a better understanding of Ca^2+^-dependent responses in *Pa* will inform about the host-pathogen relationship and help in the development of antimicrobial strategies.

Previously, we and others have collected multiple evidence illustrating the regulatory role of Ca^2+^ in *Pa*. These include Ca^2+^-induced production of virulence factors, pyocyanin (40), extracellular proteases (48, 53), biofilm formation (48, 54, 55), swarming motility, and antibiotic resistance (48, 55, 56). These virulence factors enhance colonization by *Pa* and facilitate its evasion of the host immune system (19, 57, 58). In addition, elevated Ca^2+^ negatively regulates a potent virulence factor, the type III secretion system, known to promote bacterial persistence and dissemination from the infection site (59). In the effort to elucidate the molecular mechanisms of Ca^2+^ regulation, we have identified several key components of the Ca^2+^ regulatory network in *Pa* that, among others, include the Ca^2+^-regulated two-component regulatory system CarSR (49) (identical to BqsSR in PA14 [60]), Ca^2+^ channel, CalC (not published), and Ca^2+^ sensor EfhP (40, 61).

We have established that EfhP meets all requirements of a Ca^2+^ sensor: it binds Ca^2+^ and undergoes Ca^2+^-dependent conformational changes, which are required for Ca^2+^ regulation of several aspects of *Pa* virulence (40, 61). Disruption of *efhP* leads to a decrease in Ca^2+^-regulated production of pyocyanin, and alginate, as well as *Pa* virulence in plant and *Galleria mellonella* infection models (40, 61). These data support a hypothesis that EfhP plays a key role in orchestrating the Ca^2+^ control over *Pa* gene expression. Furthermore, *efhP* is highly conserved in the *Pseudomonas* genus with even greater sequence conservation among *Pa* CF clinical isolates vs non-CF clinical and environmental isolates (61), which provides additional evidence of its role in *Pa* human infections. The predicted periplasmic localization of EfhP (61) may equip *Pa* with a mechanism to sense and respond to extracellular concentrations of Ca^2+^. Since *Pa* faces rapid transitions to millimolar levels of Ca^2+^ upon invading human airways, which are further heightened in the nasal fluids and saliva during CF (62), it is important to characterize the development of Ca^2+^ transcriptional responses over time. Therefore, we applied RNA sequencing to characterize the genome-wide kinetic changes in *Pa* transcriptome during adaptive and rapid responses to elevated Ca^2+^ and determine the role of EfhP in *Pa* transcriptional regulation by Ca^2+^.

## RESULTS

### The PAO1 kinetic transcriptional response to Ca^2+^ comprises a major share of the genome

Genome-wide RNA sequencing was conducted to identify the rapid and adaptive transcriptional changes occurring in *Pa* in response to 5 mM Ca^2+^. The selected concentration of Ca^2+^ reflects those present in the nasal fluids and saliva of CF patients, where *Pa* has been shown to undergo significant patho-adaptation (62, 63). For the designated “adaptive” response, cultures were grown to the mid-log phase (12 h) in the presence of 5 mM Ca^2+^ and compared to the mid-log 12 h cultures grown without added Ca^2+^ (Fig. S1A). For the designated “rapid” responses, PAO1 cultures were grown to the mid-log phase (12 h) in the absence of Ca^2+^ and then exposed to added 5 mM Ca^2+^ for 10 min or 60 min to be compared to the mid-log (12 h) cultures not exposed to Ca^2+^ (Fig. S1A).

**Fig 1:**
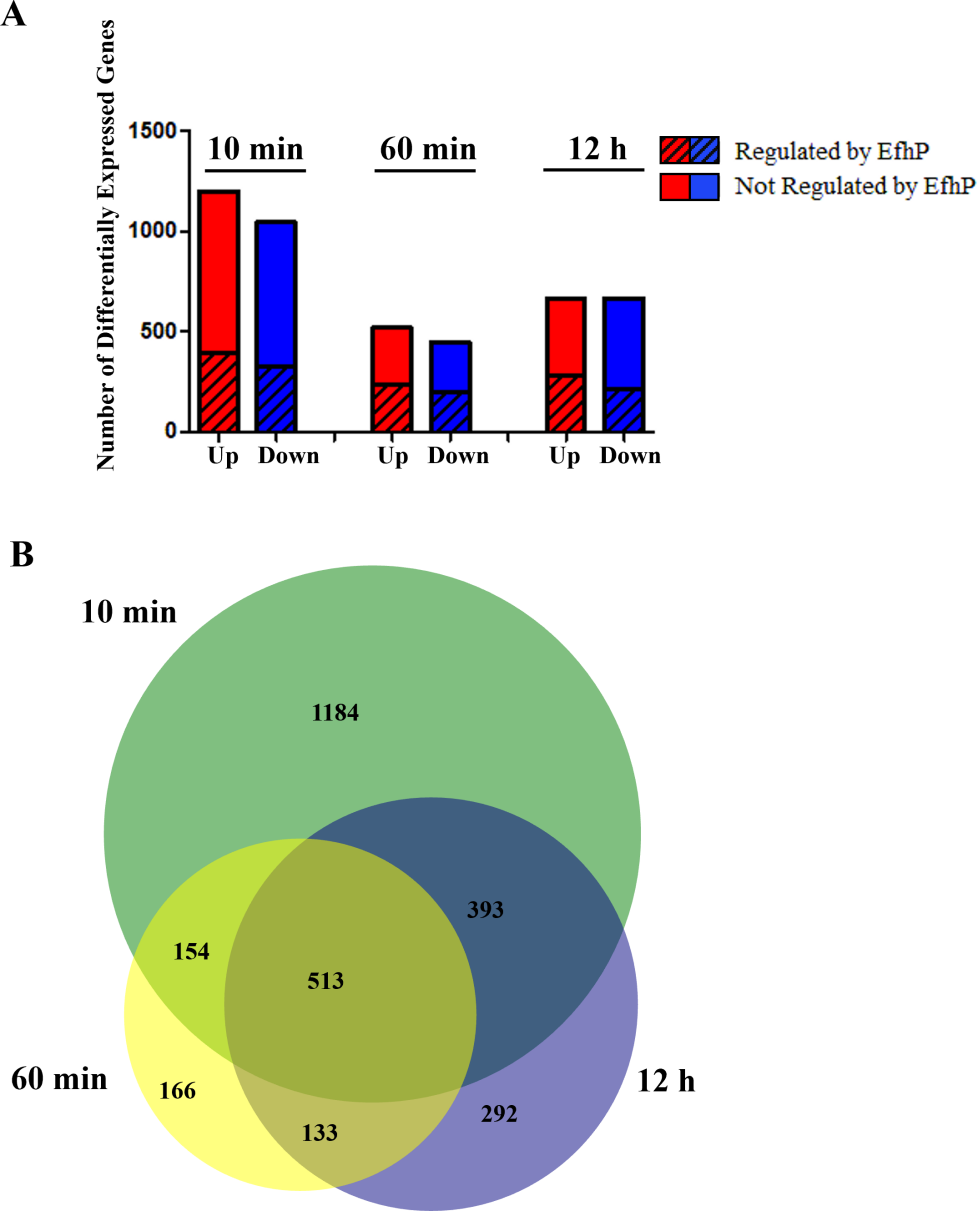
PAO1 transcriptional response to 10 min, 60 min, and 12 h exposure to Ca^2+^. (**A**) Total numbers of genes significantly upregulated (log2FC > 1, *P* < 0.05) and downregulated (log2FC < −1, *P* < 0.05) at each time point are depicted in red and blue, respectively. Dashes indicate the number of Ca^2+^-responsive genes that are regulated by EfhP at each time point. These include genes upregulated in PAO1 with a log2FC < 0 in the Δ*efhP* strain, and genes downregulated in PAO1 with a log2FC > 0 in the *ΔefhP* strain in the corresponding conditions. “Not Regulated by EfhP” indicates genes that are either significantly upregulated or downregulated in response to Ca^2+^ in both PAO1 and Δ*efhP* strains, so their regulation does not depend on the presence of EfhP. (**B**) Numbers of Ca^2+^-responsive genes (log2FC < −1 or >1, *P* < 0.05) shared by or unique to the 10 min, 60 min, and 12 h Ca^2+^ exposure conditions.

The RNAseq-based analysis of differential gene expression revealed a dynamic response to Ca^2+^ (Fig. 1A). The greatest response was detected in the 10 min condition, with 1,199 genes significantly upregulated (log2 of fold change [log2FC] > 1, *P* < 0.05) and 1,045 genes significantly downregulated (log2FC < −1, *P* < 0.05). This rapid response settled partially by 60 min of Ca^2+^ exposure (520 genes upregulated, 446 downregulated). Following 12 h adaptation to Ca^2+^, the transcriptional response increased to 665 genes upregulated and 666 downregulated. This adaptive response includes 292 unique genes not observed to be differentially regulated in the 10 min and 60 min rapid response conditions (Fig. 1B), indicating that *Pa* utilizes a specific regulatory network to adapt to long-term Ca^2+^ exposure vs short-term. Overall, of the 5,709 genes in the PAO1 genome (pseudomonas.com), 39%, 17%, and 23% were shown to be Ca^2+^-responsive in the 10 min, 60 min, and 12 h conditions, respectively (Fig. 1A and B).

**Fig 2:**
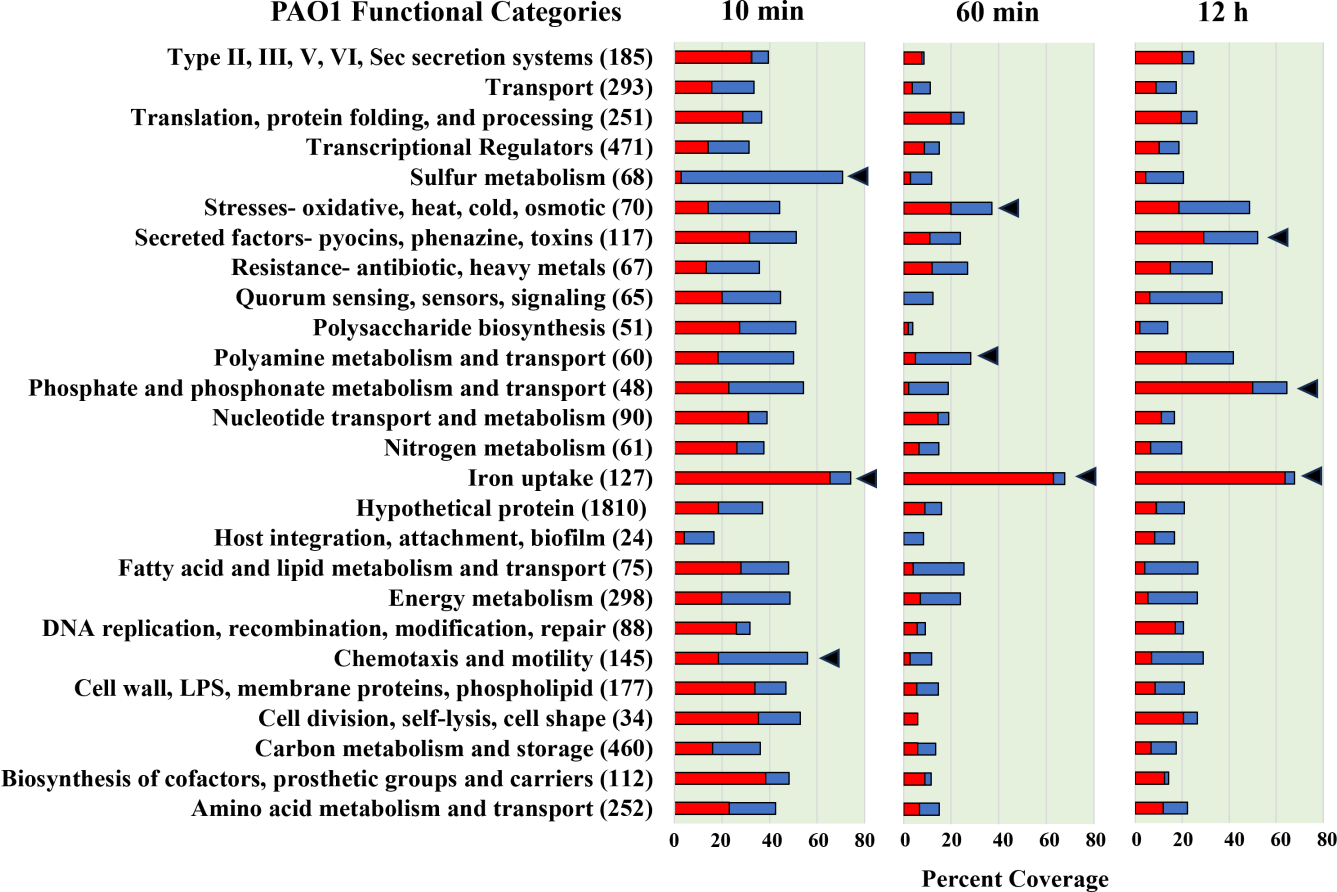
The PAO1 transcriptional response to 5 mM Ca^2+^ by functional category. RNA sequencing data from samples exposed to 5 mM Ca^2+^ for 10 min, 60 min, or 12 h was compared to samples grown in the absence of Ca^2+^. Red bars denote upregulation (log2FC > 1, *P* < 0.05) and blue bars denote downregulation (log2FC < −1, *P* < 0.05). These data present the percent coverage of each functional category which is either significantly upregulated or downregulated at each time point. The number of genes assigned to each functional category is shown in parentheses. Functional categories were selected as described in Materials and Methods. Functional categories most highly regulated by Ca^2+^ at each time point are denoted with triangles.

To gain a deeper understanding of the specific *Pa* functions regulated by Ca^2+^, the full PAO1 genome has been sorted into 26 functional categories (Fig. 2). These categories were assigned based on hand-curated gene annotation described in Materials and Methods. The percent coverage of each category was calculated by dividing the number of differentially regulated genes by the total number of annotated genes in each category. Iron (Fe) uptake was the most Ca^2+^-responsive functional category at each time point, with 74%, 68%, and 68% coverage at the 10 min, 60 min, and 12 h time points, respectively (Fig. 2). Following the Fe uptake genes, the next most Ca^2+^-responsive functional categories in the 10 min rapid response condition are sulfur metabolism and chemotaxis/motility. At 60 min exposure, Ca^2+^ regulation of these two categories is almost entirely suppressed, perhaps suggesting a regulatory correction by additional regulatory networks. However, Ca^2+^ retains a relatively high regulatory control of the Fe uptake, stresses (oxidative, heat, cold, osmotic), and polyamine metabolism/transport functional categories at 60 min (Fig. 2). In the 12 h adaptive response, phosphate, and phosphonate metabolism/transport and secreted factors (pyocins, phenazine, toxins) emerge as the most Ca^2+^-responsive genes after Fe uptake. The former two categories were also likely to be uniquely regulated by Ca^2+^ in the adaptive response, with 17 genes and 10 genes regulated, respectively. Eight genes belonging to the Hxc type II secretion system (64) were additionally upregulated during the 12 h adaptive response but not at 10 or 60 min (Fig. 2).

### EfhP regulates PAO1 rapid and adaptive responses to Ca^2+^

Previously, we have identified and partially characterized the *Pa* Ca^2+^ sensor, EfhP (40, 61). Here, we aimed to characterize the role of this protein in PAO1 transcriptional response to Ca^2+^. The presented here comparative RNA-seq analysis of Δ*efhP* grown under the same conditions as PAO1 (0 min, 10 min, 60 min, and 12 h Ca^2+^ exposure) revealed that about 31% of the total Ca^2+^ response in PAO1 requires EfhP (Fig. 1A). EfhP regulation has been called when genes upregulated (log2FC > 1) and downregulated (log2FC < −1) by Ca^2+^ in PAO1 showed Ca^2+^-dependent transcript changes of log2FC < 0 or log2FC > 0, respectively, in the mutant. This includes both rapid (10 and 60 min) and adaptive (12 h) responses (Fig. 1A). The data suggest a specific EfhP regulon within the broader PAO1 Ca^2+^ response.

### EfhP is required for Ca^2+^ regulation of iron uptake

**Fig 3:**
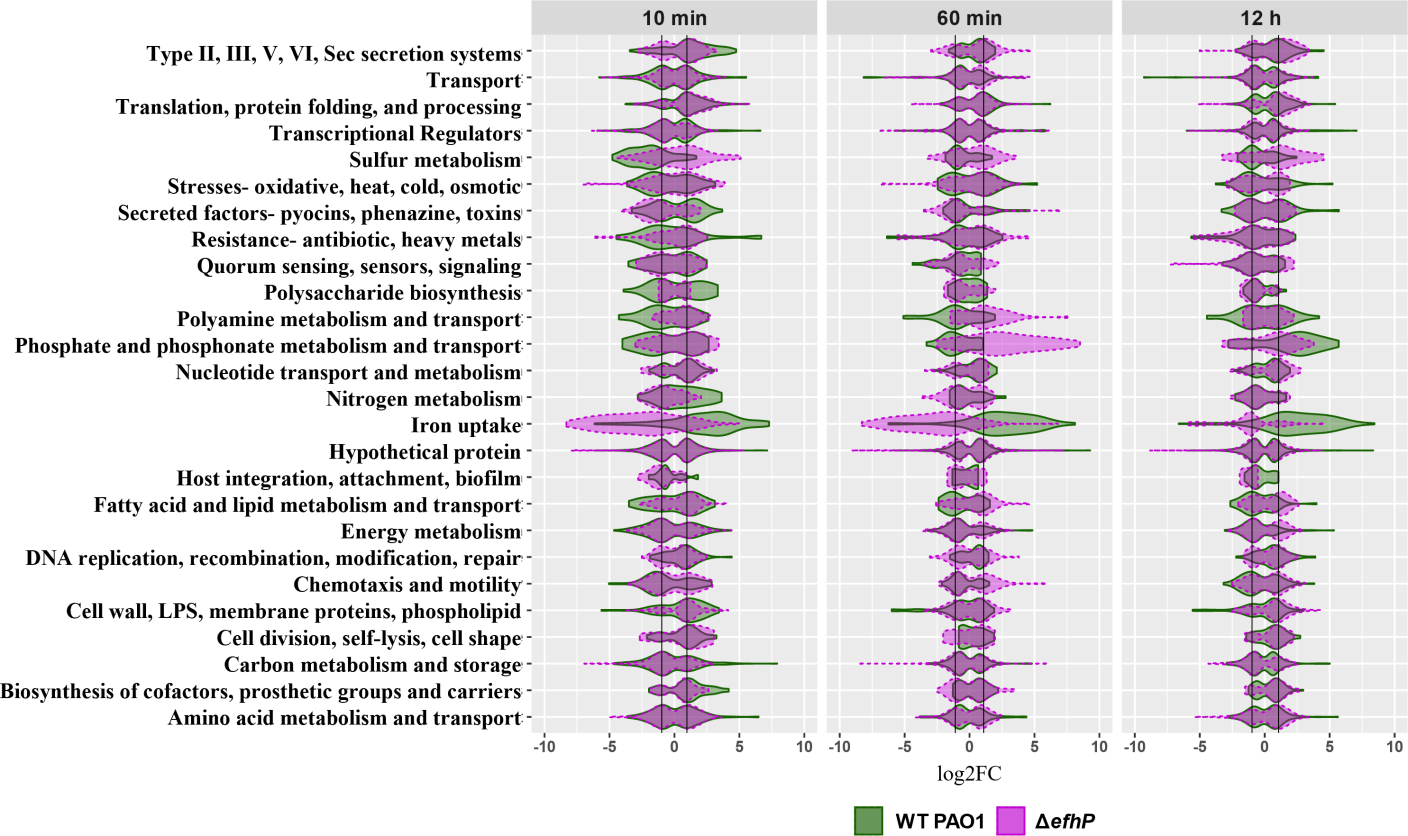
Comparing the PAO1 and Δ*efhP* transcriptional responses to 5 mM Ca^2+^ by functional category. The distributions of log2FC values for genes within each functional category are plotted for both PAO1 (green) and Δ*efhP* (purple). 10 min vs 0 min, 60 min vs 0 min, and 12 h vs 0 min condition comparisons are shown side by side. Black lines indicate log2FC = ±1. Genes falling outside of the black lines are at least twofold upregulated or downregulated. Only statistically significant genes (*P* < 0.05) were considered for the generation of the violin plots.

**Fig 4:**
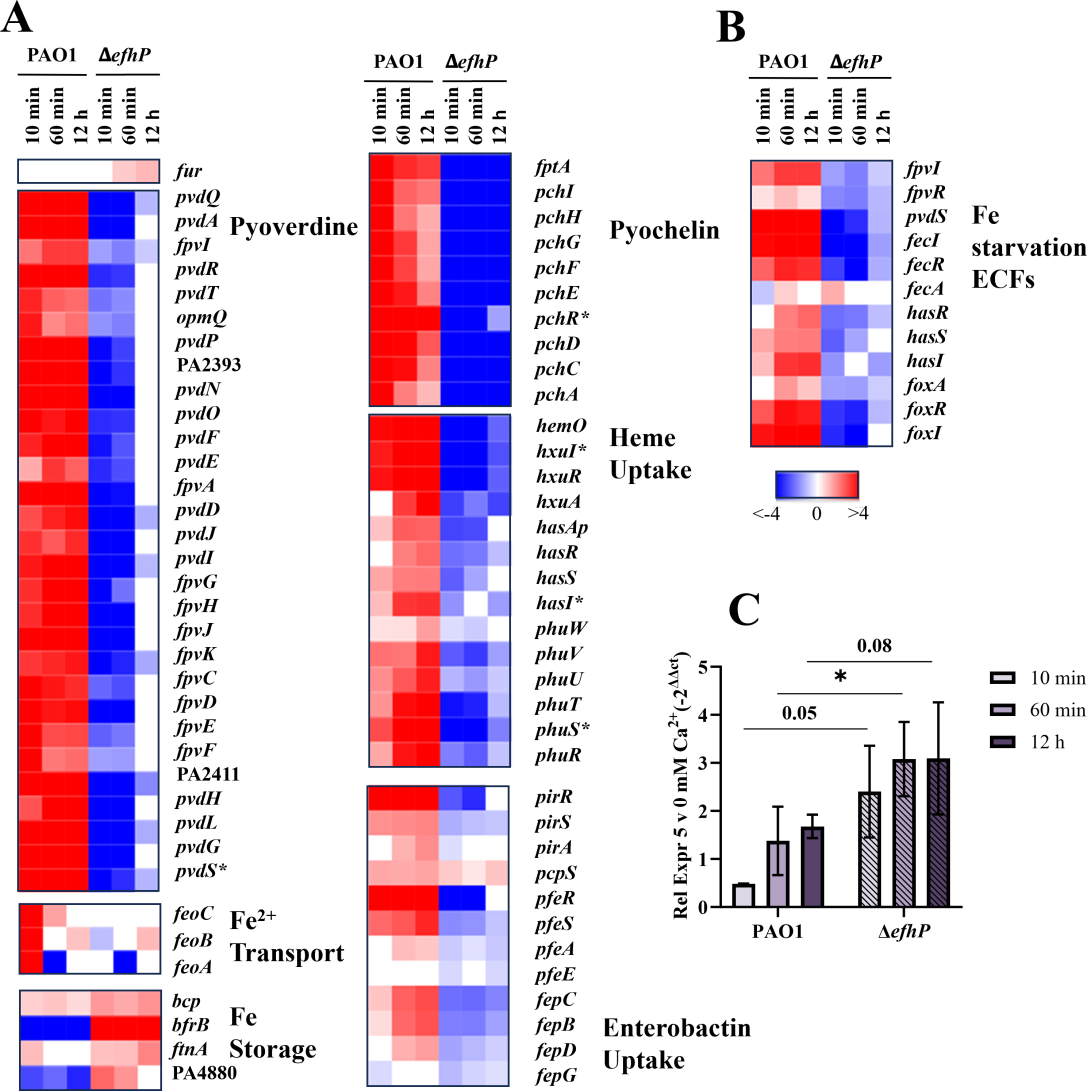
EfhP regulation of Fe uptake genes (**A**) and Fe starvation extracytoplasmic function sigma factors (ECFs) (**B**). The heat maps show log2FC values of expression comparisons of 10 min, 60 min, or 12 h exposure to 5 mM Ca^2+^ vs 0 mM Ca^2+^ condition. PAO1 and Δ*efhP* strains exposed to Ca^2+^ were compared to their respective control transcriptional profiles of cultures grown at 0 mM Ca^2+^. Major regulators are denoted with asterisks. ECF genes related to Fe starvation, as reviewed in reference 65, are shown. (**C**) Impact of Ca^2+^ on transcription of *fur* in PAO1 and Δ*efhP*. Quantitative reverse transcription-PCR (RT-qPCR) was used to evaluate the changes in *fur* transcription in PAO1 and Δ*efhP* mutant cells prepared as in panel **A**. The *efhP* mRNA levels were normalized to those of *rpoD*. Fold difference in relative transcription at 5 mM vs no Ca^2+^ was calculated using the 2^-ΔΔCt^ method. Statistical significance was determined using an unpaired one-tailed *t*-test, * indicates *P* < 0.05.

A closer examination of EfhP’s regulation within specific PAO1 functional categories revealed that the protein is required for Ca^2+^ regulation of many pathways involved in *Pa* virulence (Fig. 3). The most striking is the EfhP-dependent regulation of Fe uptake genes in both rapid and adaptive responses to Ca^2+^ (Fig. 3 and 4A). In PAO1, over 60% of the iron uptake genes are upregulated by Ca^2+^ at all three time points, whereas in Δ*efhP* these genes either do not respond or are downregulated in response to Ca^2+^. It is important to note that samples used for RNA sequencing in this study were cultured at 3.6 µM Fe, the concentration consistent with Fe levels in the CF sputum (52). So, the apparent Fe starvation response developed in the presence of Fe.

At the pathway level, the positive regulation by Ca^2+^ in PAO1 includes the genes encoding for pyoverdine, pyochelin, heme, and enterobactin uptake, along with pyoverdine and pyochelin biosynthesis genes (Fig. 4A). The Fe sequestering siderophores pyoverdine and pyochelin are known to serve critical roles in Fe acquisition from the host during *Pa* infection and contribute to virulence of the pathogen (66–68). The Has and Phu heme uptake systems are required for the utilization of heme and play a particularly significant role in *Pa* chronic infections of the CF lung (69, 70). Receptors PfeA and PirA have been established as essential for *Pa* uptake of the *Escherichia coli* siderophore enterobactin, further diversifying mechanisms of iron acquisition in this pathogen (71). A rapid upregulation of these pathways was observed after 10 min and 60 min Ca^2+^ exposure in PAO1 and extends to the 12 h exposure. In contrast, most of these genes showed downregulation in the Δ*efhP* response to Ca^2+^ (Fig. 4A). Interestingly, by 12 h Ca^2+^ exposure, transcription of most pyoverdine genes in Δ*efhP* returned nearly to the levels observed at no added Ca^2+^, but the pyochelin biosynthesis and uptake genes remain significantly down-regulated.

An opposite transcriptional profile was observed for the bacterioferritin gene *bfrB* and probable bacterioferritin PA4880, with these genes being downregulated both rapidly and adaptively in PAO1 in response to Ca^2^ but upregulated in Δ*efhP* (Fig. 4A). This stands consistent with the general Fe starvation transcriptional response (72) observed in response to Ca^2+^ in PAO1, as *bfrB* and PA4880 have previously been identified as part of Fe uptake master regulator Fur’s positive regulon, showing induction undergrowth at high Fe conditions (72, 73).

Fe acquisition systems in *Pa* are controlled by the global negative regulator Fur (74). While transcription of *fur* showed no significant Ca^2+^-responsiveness in PAO1, in Δ*efhP,* we observed 1.8-fold and 2.2-fold upregulation of *fur* following 60 min and 12 h exposure to Ca^2+^, respectively (Fig. 4A). These results were supported by quantitative reverse transcription-PCR (RT-qPCR) measurement of *fur* transcripts in PAO1 and Δ*efhP* (Fig. 4C). In agreement, according to RNA seq, the transcription of Fur-repressed *pvdS* and *pchR*, encoding important regulators of pyoverdine and pyochelin production, respectively (75, 76), was upregulated in PAO1 following 10 min, 60 min, and 12 h exposure to Ca^2+^ but downregulated in Δ*efhP* (Fig. 4A).

To sense and respond to environmental stimuli, PAO1 encodes 19 extracytoplasmic function sigma factors (ECFσ) (65). The ECFσ systems typically include transmembrane anti-σ sensors and cytoplasmic σ factors, which function to tightly regulate *Pa* stress responses (77). Interestingly, a majority of identified ECFσ in PAO1 (14 of 19) have been shown to respond to Fe starvation (65). We observed a major transcriptional induction of the Fe starvation ECFσs by Ca^2+^ in PAO1, which requires EfhP (Fig. 4B).

**Fig 5:**
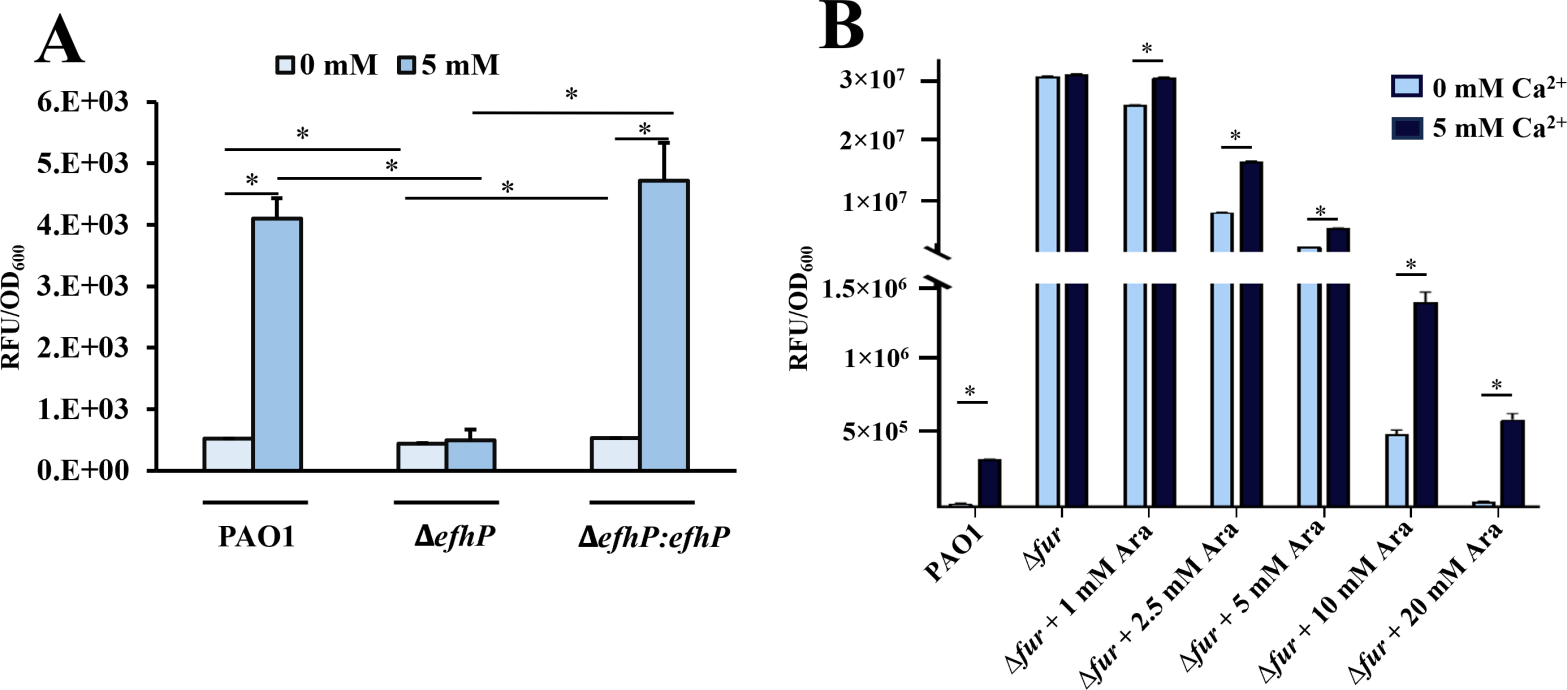
The role of EfhP (**A**) and Fur (**B**) in Ca^2+^ regulation of pyoverdine production. (**A**) Stationary (24 h) phase pyoverdine production in PAO1, Δ*efhP*, and Δ*efhP::efhP* grown in BMM8 with or without 5 mM Ca^2+^. Pyoverdine was quantified by reading fluorescence at 400 nm excitation/460 nm emission and normalized by OD_600_. (**B**) Pyoverdine production in PAO1 and arabinose-dependent conditional *fur* deletion mutant (generously shared by Dr. Imperi) (78). The mutant was cultured in the presence of varying arabinose concentrations, ranging from 0 to 20 mM to achieve increasing expression of *fur*. Pyoverdine was quantified as stated above, and the area under the kinetic curve was calculated to present the total fluorescence produced in samples during the logarithmic phase of growth. Three independent replicates were averaged, and standard error bars were depicted. Individual experiments were repeated at least three times. Statistical significance was determined by single-factor analysis of variance (ANOVA) (* indicates *P* < 0.05).

To validate EfhP regulation of *Pa* iron uptake, we have assayed for pyoverdine production by monitoring its fluorescence in PAO1, Δ*efhP,* and Δ*efhP::efhP* cultures. Supporting the transcriptional data, stationary phase (24 h) PAO1 showed a 7.8-fold increase in pyoverdine production when cultured at 5 mM Ca^2+^ compared to the 0 mM Ca^2+^ condition (Fig. 5A). This Ca^2+^ regulation of pyoverdine induction was lost in Δ*efhP* but recovered with *efhP* complementation. Since this response was observed in the cultures grown in the presence of 3.6 µM Fe in the medium, next, we aimed to characterize the impact of elevated Ca^2+^ on pyoverdine production in PAO1, Δ*efhP,* and Δ*efhP::efhP* cultures under iron starvation. Due to difficulties achieving consistent growth under prolonged iron starvation conditions, 3.6 µM added Fe was present in precultures, and main cultures were prepared in a medium with no added Fe. Interestingly, when the cultures were starved for Fe, PAO1 and Δ*efhP* pyoverdine production was shown to be insensitive to Ca^2+^ concentration (Fig. S2A), suggesting that the lack of essential Fe is higher in the hierarchy of signals than Ca^2+^. Nevertheless, pyoverdine levels normalized by OD_600_ in Δ*efhP* at both 0 mM and 5 mM Ca^2+^ concentrations were significantly lower than those observed in the wild type (Fig. S2A), indicating that EfhP is needed for a full scale of pyoverdine production under these conditions. Furthermore, Δ*efhP* growth at no added Fe was stronger than that of PAO1 and Δ*efhP::efhP* at both 0 and 5 mM Ca^2+^ (Fig. S2B), possibly due to the reduced metabolic burden of pyoverdine production in this strain. At 3.6 µM Fe, all three strains showed stronger growth at 5 mM Ca^2+^ compared to 0 mM (Fig. S2B). We also tested growth and pyoverdine production in PAO1 carrying an empty vector as a control, which had no impact. While Ca^2+^-dependent Fe uptake may partially explain the improved growth performance in the presence of Ca^2+^ and Fe, the cause of the continued growth advantage of Δ*efhP* over PAO1 and Δ*efhP::efhP* remains unknown.

To determine whether elevated Ca^2+^ leads to increased consumption of Fe causing an actual Fe starvation, we have quantified the remaining Fe in PAO1 and Δ*efhP* culture supernatants by inductively coupled plasma optical emission spectroscopy (ICP-OES). The presence of Ca^2+^ in growth medium during 10 m, 60 m, or 12 h exposure did not cause any significant reduction in Fe levels in either strain when compared to those observed at no added Ca^2+^ (Fig. S3A), supporting that Fe uptake is not increased at higher levels of Ca^2+^. It is noteworthy that the supernatants of Δ*efhP* at all Ca^2+^ conditions contained elevated Fe compared to those of PAO1 (Fig. S3A), indicating that EfhP contributes to full Fe uptake in these conditions. We also investigated whether Ca^2+^ binds pyoverdine and thus limits its availability for binding Fe. Spectral scans of pyoverdine-rich filtrates incubated in the presence of 5 mM Ca^2+^ showed slightly less pyoverdine available to bind Fe compared to 0 mM Ca^2+^ condition (Fig. S3B and C) and suggested that pyoverdine has a low affinity for Ca^2+^ as has been observed for numerous other metals (79). However, this limited effect does not seem to account for the significant changes in pyoverdine production seen at high Ca^2+^.

Finally, since Ca^2+^ regulates the entire Fe acquisition system in *Pa,* which is controlled by the negative regulator Fur (74) we hypothesized that Fur may be involved in this regulation. To test this hypothesis, we used a *fur-*conditional mutant (78) that expresses Fur upon induction with arabinose. We showed that when the expression of Fur is not induced (at no arabinose added), the production of pyoverdine in PAO1 is induced by Ca^2+^ about 1,000-fold vs that in the WT at no Ca^2+^ (Fig. 5A and B). This agrees with its negative regulation of *pvd* genes. In addition, we observed no Ca^2+^ induction in the *fur* mutant. However, when increased amounts of arabinose were added to the cultures (1–20 mM) to induce the expression of *fur* (78), the level of pyoverdine decreased back to the level in PAO1, and the induction of Ca^2+^ was restored (Fig. 5B). These results suggest that Ca^2+^ regulation of pyoverdine production involves Fur. The mechanisms of this involvement will be the focus of future studies.

### EfhP is required for Ca^2+^ regulation of pyocins and self-lysis

**Fig 6:**
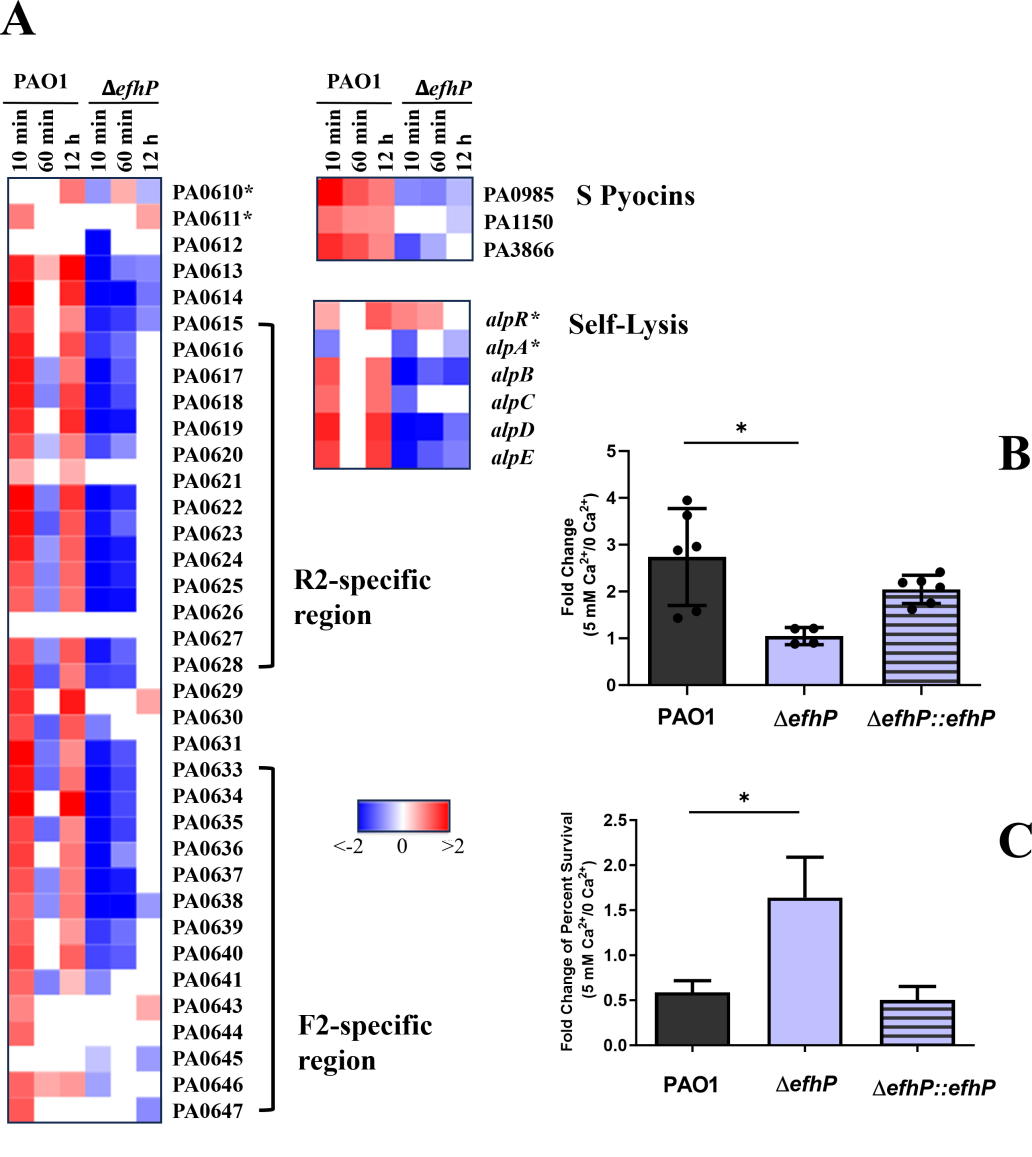
EfhP regulation of pyocin and self-lysis genes. (**A**) The heat maps show log2FC values of expression comparisons of 10 min, 60 min, or 12 h 5 mM Ca^2+^ conditions vs 0 mM Ca^2+^ conditions. PAO1 and Δ*efhP* strains were compared to their respective control transcriptional profiles of cultures grown at 0 mM Ca^2+^. Pyocin and self-lysis regulators are denoted with asterisks. (**B**) Impact of Ca^2+^ on transcription of *prtN*. The gene expression of *prtN* in WT, ∆*efhP*, and ∆*efhP::efhP* during mid-log was evaluated using RT-qPCR. The relative mRNA levels were normalized to the reference gene, *nadB*. The impact of Ca^2+^ on transcription was determined by calculating the ratio of *prtN* expression at 5 mM Ca^2+^ vs no Ca^2+^. Statistical significance was determined using one-way ANOVA. * indicates *P* < 0.05. (**C**) Impact of Ca^2+^-induced pyocin production on the survival of R-type pyocin-sensitive *Pa* strain 13S. A 100 µL of 13S culture (OD_600_ 0.3) was treated with 100 µL of supernatants collected from PAO1, Δ*efhP*, and Δ*efhP::efhP* grown in 0 or 5 mM Ca^2+^ BMM8. The fold change of 13S survival upon the addition of respective supernatants from 5 vs 0 mM Ca^2+^ cultures of wild-type PAO1, Δ*efhP*, and Δ*efhP::efhP* was calculated by dividing percent survival at 5 mM Ca^2+^ by that at no Ca^2+^. Treatments were performed in triplicate, and the experiments were repeated independently three times. An unpaired one-tailed *t*-test was used to determine statistical significance (* indicates *P* < 0.05).

EfhP’s regulation of the *Pa* response to Ca^2+^ extends to pathways involved in pyocin production and self-lysis (Fig. 6A). The *Pa* genome encodes three different types of bacteriocins (known as pyocins), which have bactericidal activity against same or closely related *Pseudomonas* species (80). This includes the R- and F-type pyocins, whose genes are clustered within a chromosomal locus in PAO1, and the S-type pyocins, which have a more scattered distribution across the genome (80). In this study, upregulation in PAO1 transcription of R- and F-type pyocins was observed following 10 min and 12 h exposure to Ca^2+^, while S-type pyocins were upregulated at all three conditions (Fig. 6A). This Ca^2+^-dependent induction of most pyocin genes was nearly absent in Δ*efhP* at all time points. Additionally, most of the *alp* self-lysis genes were upregulated in the PAO1 response to Ca^2+^ at 10 min and 12 h but downregulated in Δ*efhP*. Interestingly, transcription of regulators *alpR* and *alpA* was unaffected in the Δ*efhP* response to Ca^2+^, but Ca^2+^ exposure repressed much of their regulon, *alpBCDE* (Fig. 6A).

To validate the observed Ca^2+^ regulation of pyocin biosynthesis, we first analyzed the expression of the positive transcriptional regulator for R-type pyocin biosynthesis, *prtN* (81), by using RT-qPCR. Supporting the RNA-seq data, the expression of *prtN* increased in response to elevated Ca^2+^ in WT, remained unchanged in Δ*efhP,* and was partially complemented in Δ*efhP::efhP* strain (Fig. 6B). In addition to this, we have also tested the impact of Ca^2+^-induced R-type pyocin production by measuring the survival of R-type pyocin-sensitive *Pa* strain 13S (82). This clinical isolate has been specifically selected for its sensitivity toward the R-type pyocins (83, 84). To do this, the 13S strain was grown to the middle log in BMM8, and the cultures normalized to OD_600_ of 0.3 were treated with the supernatants obtained from the PAO1, Δ*efhP,* and Δ*efhP::efhP* grown in the presence or absence of 5 mM Ca^2+^. The calculated fold differences in survival of 13S upon treatments with Ca^2+^-induced and Ca^2+^-non-induced supernatants of the three strains are shown in Fig. 6C. In agreement with the transcriptomics data, the presence of Ca^2+^ during PAO1 growth resulted in the supernatants that reduced 13S survival two times (fold change 0.5). This relationship was inversed in the case of Δ*efhP,* where we observed 1.6-fold increased survival, and reversed back to the WT level in Δ*efhP::efhP*. These data support the RNA-seq data suggesting Ca^2+^ induction of R-type pyocin production in PAO1, which requires the presence of EfhP.

### The expression of *efhP* is regulated by Ca^2+^, Fe, growth phase, and the two-component system CarSR

**Fig 7:**
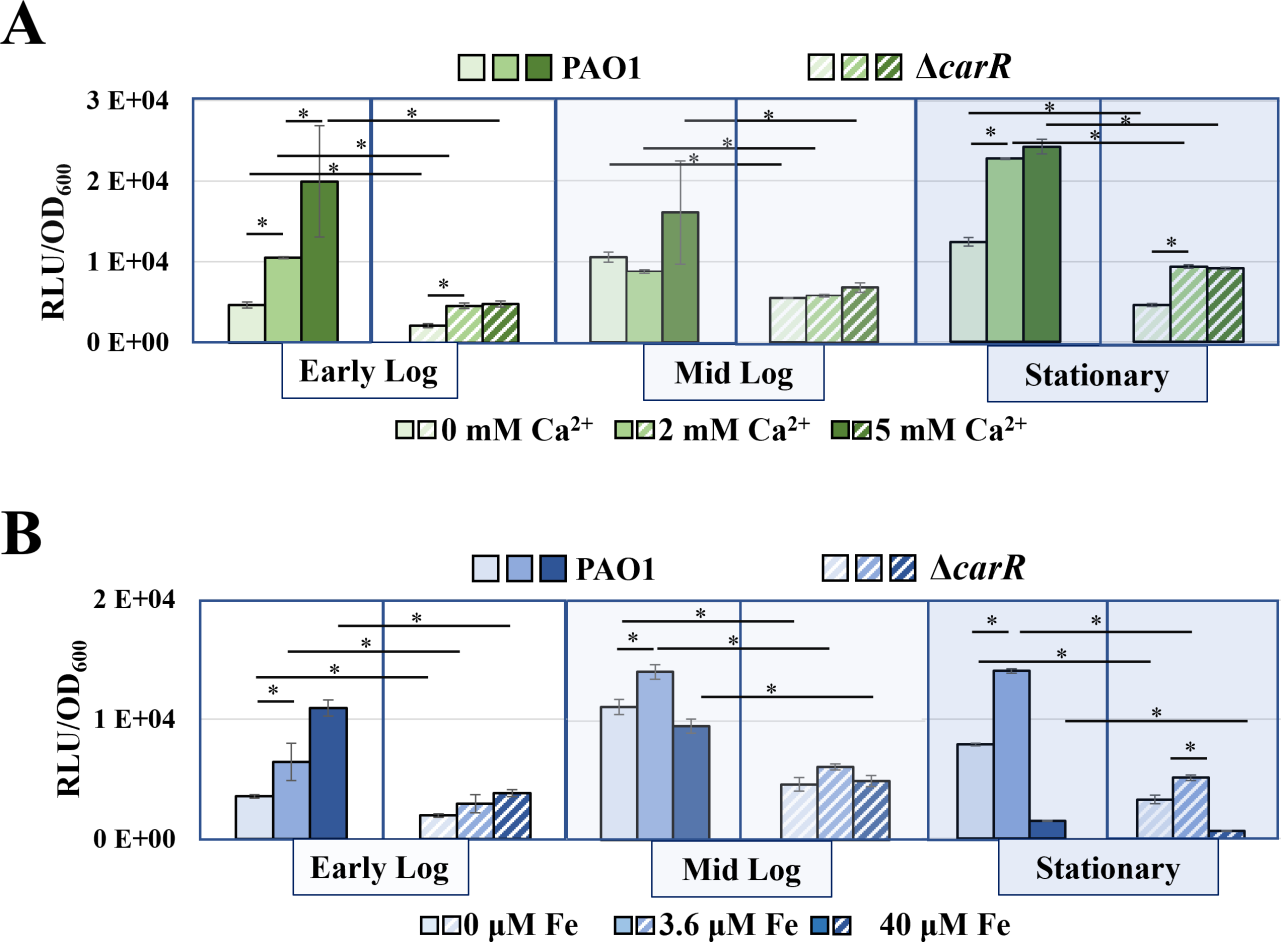
Impact of elevated levels of Ca^2+^ and Fe on *efhP* transcription. The wildtype PAO1 (solid bars) and Δ*carR* (dashed bars) harboring the *efhP* promoter construct were grown at no or elevated levels of Ca^2+^ (**A**) or Fe (**B**). Luminescence normalized by OD_600_ is plotted at singular time points to represent early log (4 h), mid-log (6–7 h), and stationary (12 h) for each strain and condition. Graphs represent the mean of at least three biological replicates and error bars indicate the standard error. Statistical analysis was done by univariate ANOVA in SPSS (version 29.0.0.0, IBM Corp. 2022) with a confidence interval of 95% (* indicates *P* < 0.05).

The RNA-seq analysis revealed that the expression of *efhP* was slightly increased after 12 h of PAO1 growth in the presence of Ca^2+^ but was not responsive to the ion after shorter exposures. Since this change was not statistically significant, we aimed to generate additional insight and tested the impact of 2 mM and 5 mM Ca^2+^ on *efhP* expression during different phases of growth by using a promoter fusion assay. For this, the *efhP* promoter was fused to a promoterless *luxCDABE* reporter, which upon activation produces luminescence. Monitoring the resultant normalized luminescence informs about the transcriptional activation of the promoter. It was observed that the presence of Ca^2+^ caused an increase in transcription of *efhP* during both the early log and stationary phases of growth but not in the mid-log phase (Fig. 7A).

Considering the discovered role of Ca^2+^ in regulating Fe uptake, we also aimed to characterize the potential role of Fe in regulating the transcription of *efhP*. For this, we evaluated the promoter activity of *efhP* during growth of PAO1 at no added or provided with 3.6 µM or 40 µM Fe in the absence of Ca^2+^. Although the overall impact was smaller than that in response to Ca^2+^, the *efhP* promoter activity was also regulated by Fe (Fig. 7B). However, in this case, we observed an increase in response to 3.6 µM at all the time points and a decrease at 40 µM Fe in both mid-log and stationary phase cells.

Others have reported the regulation of *efhP* transcription by the two-component system BfmRS, which was also shown to control *Pa* biofilm production (85, 86). We have chosen to investigate the regulation of *efhP* by another *Pa* two-component system, CarSR (60). Our previous work showed that CarSR is regulated by Ca^2+^ and positively regulates the expression of several genes, including *carO* and *carP* in a Ca^2+^-dependent manner (49). An identical homolog of CarR in PA14, BqsR has been shown to be regulated by Fe (60). This indicates that CarR may mediate the regulatory impact of elevated levels of Ca^2+^ and Fe on the transcription of *efhP*. Hence, we measured the *efhP* promoter activity in the Δ*carR* mutant in response to elevated Ca^2+^ and Fe. Supporting the regulatory role of CarR, promoter activity of *efhP* in Δ*carR* background was significantly lower than that in PAO1 at most of the tested conditions with the strongest difference during early log and stationary growth phases (Fig. 7A and B).

### *efhP* expression is elevated in CF clinical isolates

It is important to understand whether *efhP* plays a role in *Pa* adaptation to the CF lung. Supporting such a role, the reported microarray data showed that the transcription of *efhP* is greatly increased in clinically relevant conditions. For example, an *in vivo* microarray study utilizing sputum from the lungs of CF patients detected that *efhP* belongs to the top 1% of the most highly expressed *Pa* genes during infection (87). Additionally, *efhP* expression was shown to be greatly elevated in a CF clinical isolate exhibiting mucin sulfatase activity (88) as well as in later stages of infection with *Pa* (89).

**Fig 8:**
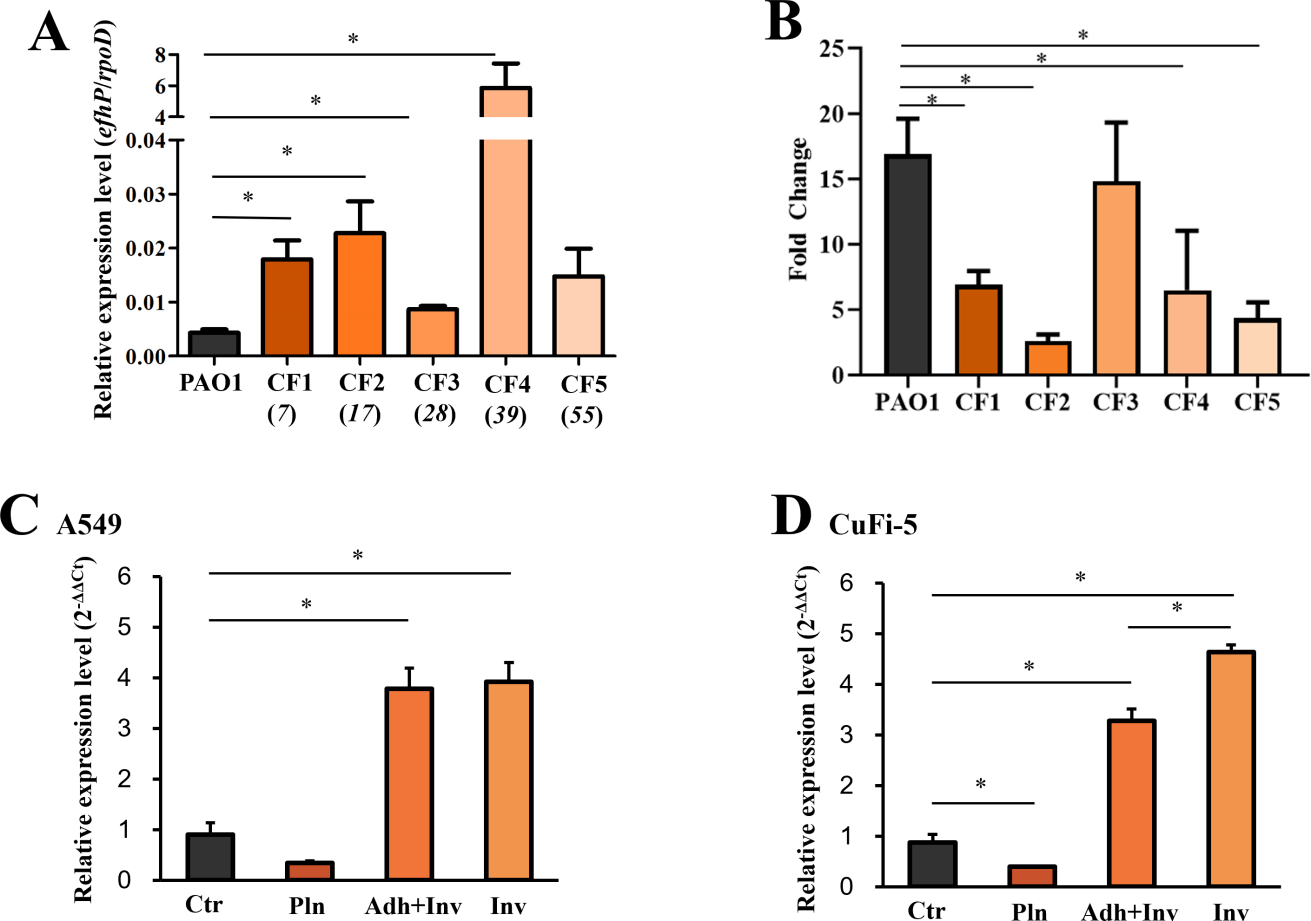
Analyses of *efhP* regulation (**A**) and pyoverdine production (**B**) in CF clinical isolates and *efhP* regulation during *Pa* interactions with A549 epithelial cells (**C**) and CuFi-5 epithelial cells (**D**). (**A**) Mid-log *efhP* transcription by five CF clinical isolates grown in Luria–Bertani (LB) medium was compared to that of PAO1 by RT-qPCR. The ages of CF patients from which each isolate originated are indicated in italics. The relative mRNA levels were normalized using a standard curve as described in Materials and Methods. Statistical significance between clinical isolates vs PAO1 was determined by single factor ANOVA (* indicates *P* < 0.05). (**B**) Pyoverdine production in CF clinical isolates grown in BMM8 with or without 5 mM Ca^2+^. Pyoverdine was quantified by reading fluorescence at 400 nm excitation/460 nm emission and normalized by OD_600_. The area under the curve was calculated to present the total fluorescence produced during the logarithmic phase of growth. Statistical significance was determined by single-factor ANOVA (* indicates *P* < 0.05). Transcription of *efhP* during *Pa* interactions with A549 (**C**) or CuFi-5 (**D**) epithelial cells. RT-qPCR was conducted using RNA obtained from growing PAO1 without epithelial cells (Ctr), planktonic (Pln), adhered+invaded (Adh+Inv), or invaded (Inv) PAO1. The relative mRNA levels were normalized to those of *rpoD,* and the fold difference was calculated using the 2^-ΔΔCt^ method. Median average and standard errors from three biological replicates are shown. For statistical analysis, both one-way ANOVA and Tukey-Kramer tests were performed (* indicates *P* < 0.05).

To evaluate the levels of *efhP* expression in CF clinical isolates, we selected sputum *Pa* isolates from CF patients ranging in age from 7 to 55 years and tested for *efhP* expression via RT-qPCR. The different ages were selected for potential correlation between the level of *efhP* expression and the advancement of disease. Most CF isolates showed at least twofold increased *efhP* expression in comparison to that in PAO1, with one of them (CF4) expressing *efhP* at the level 1,484-fold above that of PAO1 (Fig. 8A). While no clear trends in *efhP* expression emerged in relationship with the ages of CF patients, a larger sample of isolates may aid this effort in future studies.

Based on the observed high level of *efhP* expression in the selected set of *Pa* CF clinical isolates, we hypothesized that the pyoverdine production in these isolates is increased in response to elevated Ca^2+^. To test this hypothesis, five selected isolates were grown in the presence or absence of 5 mM Ca^2+^, and their pyoverdine production was evaluated based on its OD_600_-normalized fluorescence. As expected, we observed a significant Ca^2+^-dependent increase in pyoverdine levels in all five isolates (fold difference in response to Ca^2+^ shown in Fig. 8B). These data substantiate the regulatory role of Ca^2+^ for Fe uptake in clinical *Pa* strains.

### The expression of *efhP* is regulated by interactions with host cells

Considering the reported and observed high levels of *efhP* expression in clinically relevant samples, we hypothesized that the expression of the gene is elevated during *Pa* interactions with host cells. To monitor the regulation of *efhP* expression during interactions of PAO1 with host cells, two types of human lung epithelial cell lines (adenocarcinoma alveolar basal epithelial cells A549 and CF bronchial CuFi-5 epithelial cells) were infected with PAO1. Following the infection, two experimental and two control populations of PAO1 were collected for RT-qPCR. The experimental populations included PAO1 adhered to epithelial cells and those internalized/invaded them. Since adhered PAO1 cells could not be separated from invaded cells, this group was considered a mixture. As controls, we used planktonic PAO1 (free-swimming above the epithelial cells) and bacteria incubated alone. The quantities of the collected bacteria in each category are summarized in Table S1. The results revealed at least a threefold increase in *efhP* expression upon interaction with host cells (Fig. 8C and D). The increase in the *efhP* transcription during invasion is stronger in CuFi-5 cells when compared to A549 cells.

## DISCUSSION

**Fig 9:**
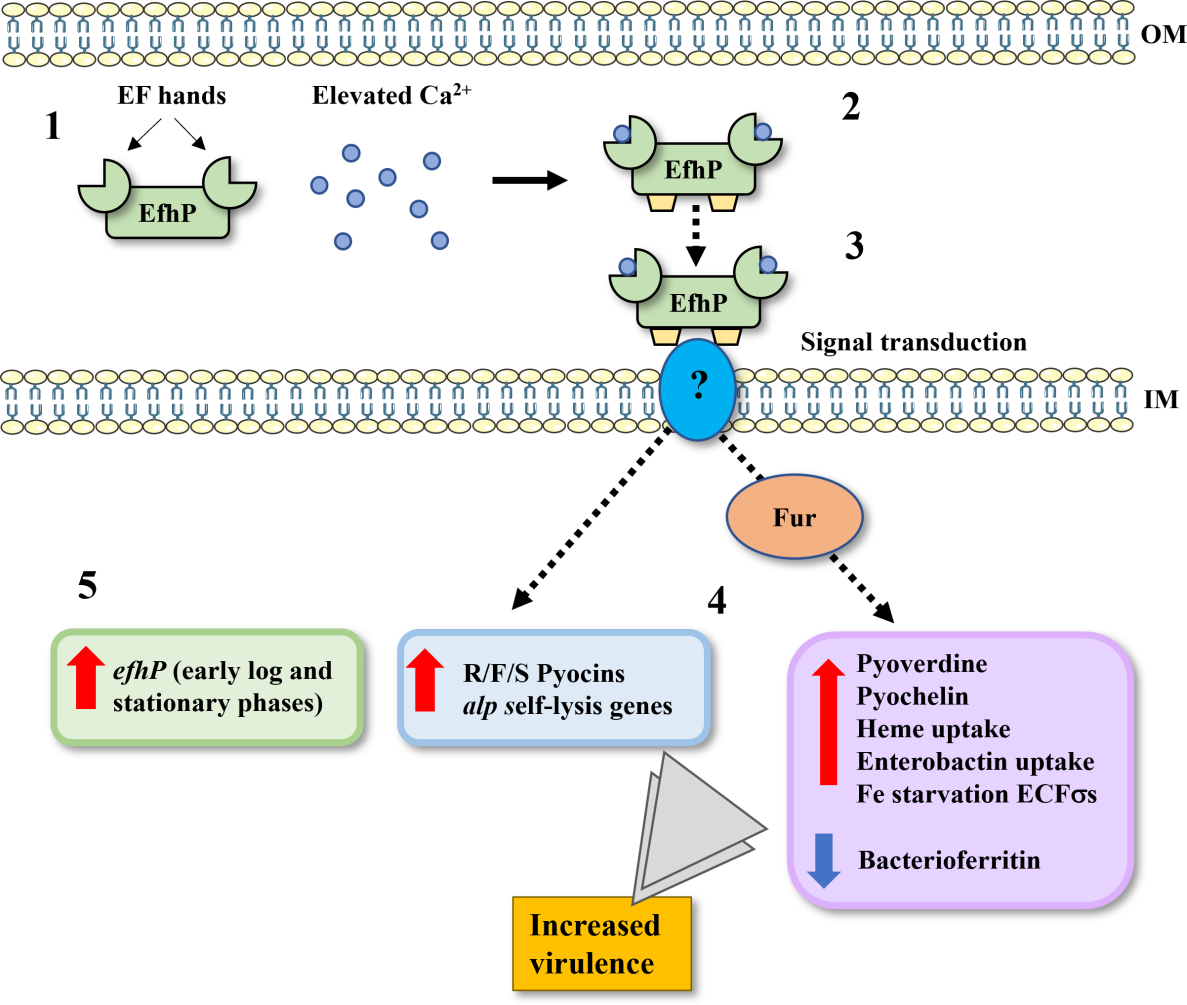
Summary of the previously reported and presented data illustrating the role of EfhP in the *Pa* response to Ca^2+^. (1) Apo-EfhP is predicted to reside in the periplasm, where it can be exposed to elevated levels of Ca^2+^. (2) Upon binding of Ca^2+^ with its EF hands, EfhP undergoes conformational changes, exposing hydrophobic regions of the protein (shown in yellow) (61). (3) It is hypothesized that these conformational changes allow EfhP to interact with still-unknown protein partners and transduce the Ca^2+^ signals. (4) Rapid and adaptive transcriptional changes take place, as revealed by RNA-seq analysis. Increases to the transcription of pyocin and self-lysis genes and the Fur regulon of Fe starvation genes are EfhP-dependent. Additionally, the downregulation of select Fe storage genes (bacterioferritin) takes place at elevated Ca^2+^ in an EfhP-dependent manner. Together, this regulation of Fe starvation and pyocin/self-lysis by Ca^2+^ and EfhP is expected to increase *Pa* virulence in the host. EfhP’s role in Ca^2+^-induced *Pa* virulence has been previously established in lettuce leaf and *G. mellonella* (40, 61) infection models. (5) Promoter fusion data has revealed regulation of *efhP* transcription at elevated Ca^2+^ levels that are partially dependent on CarR. This suggests the presence of other *Pa* mechanisms for sensing of Ca^2+^, responsible for regulating the transcription of *efhP* and other EfhP-independent genes within the *Pa* Ca^2+^ response.

Previous studies have identified Ca^2+^ regulation of numerous *Pa* virulence factors (48, 49, 90) contributing to increased mortality in *in vivo* infection models (50, 61, 91). We have identified the EF-hand Ca^2+^ sensor, EfhP, that binds Ca^2+^ and controls several *Pa* Ca^2+^ responses, including the induction of pyoverdine (40) and pyocyanin production (61), and elevated virulence in lettuce leaf and *G. mellonella* infection models (40, 61). In this study, we utilized RNA sequencing analysis to characterize the *Pa* rapid and adaptive transcriptional responses to Ca^2+^ which potentially drive the observed increases in virulence. Most notably, the results revealed a strong regulatory link between Ca^2+^ signaling and Fe sequestering mechanisms. Numerous Fe uptake pathways in PAO1 were induced by Ca^2+^, including the biosynthesis and uptake of siderophores pyoverdine and pyochelin, and this regulation requires EfhP. Transcription of *efhP* itself is shown to be tuned by both Ca^2+^ and Fe, significantly elevated in *Pa* clinical isolates, and increased by interactions with host cells. The previously reported discoveries are summarized in Fig. 9 and support the anticipated roles of EfhP in the *Pa* responses to the host-related factor Ca^2+^ benefiting its virulence and adaptation to the host.

Global RNA seq analysis indicated a capacity of PAO1 for kinetic transcriptional responses over the course from 10 min to 60 min to 12 h of Ca^2+^ exposure. While the Fe uptake genes account for a large share of Ca^2+^ responsive genes at all three-time points, other functional categories made up more specific kinetic responses. These include sulfur metabolism and chemotaxis/motility genes in the rapid response to Ca^2+^, and secreted factors (pyocins, phenazines, toxins) and phosphate/phosphonate metabolism/transport genes in the adaptive response. The timing of these responses potentially reflects the functional significance of the corresponding processes for rapid and long-term adaptation strategies to environments with elevated Ca^2+^. Interestingly, the 10-min condition shows the greatest number of differentially expressed genes, indicating that sensing Ca^2+^ triggers a massive transcriptional response and emphasizing the important regulatory roles of rapid Ca^2+^-sensing.

In addition to equipping *Pa* with Fe sequestering mechanisms, Ca^2+^ regulation of Fe homeostasis may also have implications for the multitude of Fe-controlled virulence factors. Production of the Fe^3+^-sequestering siderophores pyoverdine and pyochelin itself has been associated with increased *Pa* virulence (66, 92, 93). Furthermore, the production of biofilm and extracellular endoprotease PrpL (48) are regulated by both Fe (94, 95) and Ca^2+^, supporting the regulatory overlap of the signaling cascades (48). Co-regulation of virulence by Fe and Ca^2+^ may have especially significant impacts on the pathogenicity of *Pa* in the CF lung where free Ca^2+^ (62) was shown to be elevated vs those seen in healthy individuals.

The RNA seq analysis showed that EfhP is required for 31% of the total PAO1 Ca^2+^ response, indicating the involvement of other Ca^2+^ signaling and regulatory systems, possibly including Ca^2+^-dependent two-component system CarSR (49) and Ca^2+^ sensor LadS (90). Fe uptake pathways including pyoverdine, pyochelin, heme, and enterobactin as well as Fe storage fell under the regulatory control of EfhP at all the Ca^2+^ exposure intervals. The role of EfhP in regulating adaptive Fe uptake was supported by previous proteomic analysis conducted in PAO1 and mucoid strain FRD1 grown both planktonically and in biofilms (40). Deletion of *efhP* from these strains abolished or significantly reduced the Ca^2+^-dependent expression of several Fe uptake proteins, including pyoverdine biosynthesis proteins PvdNO, Fe^3+^-pyochelin outer membrane receptor precursor FptA, Fe^3+^-binding periplasmic protein HitA, and a putative binding protein of iron ABC transporter PA5217 (40). In agreement, in the present analysis, all the encoding genes were transcriptionally regulated by EfhP at 5 mM Ca^2+^.

The observed Ca^2+^ induction of Fe uptake was not a result of Fe deficiency, as illustrated by ICP-OES-based total Fe quantification in PAO1 and Δ*efhP* strains cultured under the conditions used for RNA sequencing. The presence of elevated Ca^2+^ did not increase Fe uptake even after 12 h Ca^2+^ exposure. This supports the hypothesis that the regulation of Fe uptake genes by Ca^2+^ is a result of Ca^2+^ signaling mechanisms rather than lack of Fe. Interestingly, Δ*efhP* showed significantly less Fe uptake at all Ca^2+^ conditions compared to PAO1. Taking into account the increased growth of Δ*efhP* at no Fe compared to PAO1, it appears that EfhP’s contributions to full Fe uptake became a growth hindrance when no Fe is present.

Another possible mechanism of Ca^2+^ interference with Fe-signaling cascades is via direct interaction with *Pa* siderophores. In addition to Fe^3+^, pyoverdine was shown to chelate 16 other metals at lower efficiencies, including Ga^3+^, Cu^2+^, and Ni^2+^ (79). Ga^3+^ disrupts *Pa* Fe homeostasis via competition with Fe^3+^ for binding to pyoverdine, from which it is unable to be reduced and released once in the periplasm (79, 96, 97). Cu^2+^, in addition to binding pyoverdine, was shown to stimulate pyoverdine production (79, 98), possibly via the generation of oxidative stress. In this study, we show that 5 mM Ca^2+^ has a slight repressive effect on pyoverdine-Fe complex formation, likely due to a low pyoverdine affinity for Ca^2+^. While the ability of pyoverdine to bind Fe is partially hindered by elevated Ca^2+^, it appears that this impact is too subtle to cause Fe starvation.

In biological systems, Fe is present in one of its two distinct oxidation states, ferrous (Fe^2+^) or ferric (Fe^3+^) (99), with the ferric form being most abundant in aerobic conditions (100). *Pa* uses siderophores such as pyoverdine and pyochelin to bind Fe in its ferric state (101) which is reduced to its ferrous state and unloaded from pyoverdine in the periplasm. Once released from pyoverdine, Fe^2+^ enters the cytoplasm and binds the ferric uptake regulator (Fur), which regulates genes involved in Fe homeostasis, uptake, and storage and thus controls the responses to Fe availability (78, 99). It is currently unknown whether elevated intracellular Ca^2+^ concentrations, typically tightly regulated in *Pa* (102), have an effect on Fe^2+^ binding to Fur and other proteins. Deletion of *efhP* was shown to significantly disrupt intracellular Ca^2+^ homeostasis (40), providing a possible mechanism by which the Ca^2+^ sensor enacts regulatory control. While Fur is recognized as the master regulator of Fe uptake and is a conditionally essential protein in *Pa* (78), it does not act alone. Fur only indirectly regulates pyoverdine biosynthesis, acting as a repressor of σ factor *pvdS* under Fe-replete conditions (103, 104). When Fur binds Fe^2+^, it releases from the *pvdS* promoter region, allowing *pvdS* expression. PvdS then binds anti-σ FpvR, from which it is released upon PVD-Fe binding to receptor FpvA, resulting in transcription of the pyoverdine biosynthetic genes (103, 105, 106). EfhP’s predicted presence in the periplasm would allow it to interact with components of the Fe responsive ECFσ systems, such as FpvARI/PvdS, FecARI, HasRSI, and FoxARI which are transcriptionally regulated by Ca^2+^ in an EfhP-dependent manner as presented in this RNA seq analysis. Interaction between EfhP and a Fe starvation ECFσ at elevated Ca^2+^ could have strong effects on the transcription of Fe uptake genes considering the high degree of positive feedback following activation of these systems (107, 108). In Fig. 9, we present a schematic summary of the previously reported and present evidence supporting the role of EfhP in mediating the *Pa* Ca^2+^ response, including Fe uptake.

Direct sensing of Fe^3+^ or Fe^2+^ by EfhP is an additional possibility. The recent discovery of conserved cysteine residues within four repeats of “EGKCG” in the EfhP sequence has led to the hypothesis that EfhP may serve dual Ca^2+^ and Fe-sensing functions. While these specific repeats have not yet been characterized, cysteine residues are known to serve Fe-sensing roles in other proteins (109, 110). Another example is the four Cys residues in FeoC, shown to bind a redox-active Fe-S cluster and interact with FeoB, responsible for passing Fe^2+^ (111–113). Potential dual Ca^2+^ and Fe-sensing by EfhP would help explain the observed high degree of crosstalk between the two signaling cascades and is the current focus of our research. We are also investigating the mechanisms of EfhP signal transduction following a hypothesis that upon sensing Ca^2+^ (Fe) EfhP interacts with proteins (e.g., anti-sigma factors) that relay the signal(s) toward transcriptional regulation.

In this study, we have identified the transcriptional regulation of *efhP* itself by Ca^2+^ and Fe, which requires the two-component system CarSR (BqsSR). CF sputum contains about four times higher levels of Fe (114) and two times higher levels of Ca^2+^ (62) as compared to that in healthy individuals. Hence, it is possible that the elevated levels of Fe and Ca^2+^ in the host environment regulate the expression of *efhP* during the infection process, as was observed in the promoter-reporter data. While the presence of Fe promoted the expression of *efhP* in the early log phase, higher levels of Fe acted as a repressor of *efhP* expression in mid-log and stationary phases, suggesting additional growth phase-dependent regulation. Elevated Ca^2+^ had a positive regulatory effect on *efhP* expression in both early log and stationary phases. Increased transcription of *efhP* is thus identified as part of the *Pa* response to both Ca^2+^ and Fe, adding a new dimension to its regulation of Fe uptake at high Ca^2+^ as revealed by the RNA seq analysis.

Finally, we sought to further understanding of *efhP* expression during infection. Microarray expression data from the GEO database showed that the transcription of *efhP* is >150-fold higher in *Pa* isolates from CF sputa grown both *in vivo* and *in vitro* (GDS2869 and GDS2870), indicating the importance of this gene during infection and suggesting that its expression is responsive to the host environment. Here, we showed that all five selected CF isolates express *efhP* at levels higher than PAO1 *in vitro*. Additionally, we showed that expression of *efhP* is increased by *Pa* interaction with host epithelial cells. Upon invasion of host cells, *Pa* transitions from millimolar levels of Ca^2+^ in the extracellular space to nanomolar levels of free cytosolic Ca^2+^ (45). The transcription of *efhP* increases following invasion despite the lowered Ca^2+^, suggesting that EfhP may have a low-Ca^2+^ mode of action as well, potentially regulated by stronger, non-Ca^2+^ transcriptional triggers, which enables EfhP’s role in *Pa* intracellular survival. Together with the previously demonstrated impact of EfhP on virulence in various infection models, it is expected that the Ca^2+^ sensor plays a significant role in *Pa* adaptation and virulence during infection of the human host.

## MATERIALS AND METHODS

### Bacterial strains, plasmids, and media

Table 1 lists the bacterial strains and plasmids used in this study. Biofilm minimal medium (BMM) (48) was used to grow *Pa* strains. BMM consists of 9.0 mM sodium glutamate, 50 mM glycerol, 0.02 mM MgSO_4_, 0.15 mM NaH_2_PO_4_, 0.34 mM K_2_HPO_4_, 145 mM NaCl, 200 µL trace metals, and 1 mL vitamin solution (per liter of medium). The trace-metals mixture was prepared with 5.0 g CuSO_4_·5H_2_O, 5.0 g ZnSO_4_·7H_2_O, 5.0 g FeSO_4_·7H_2_O, and 2.0 g MnCl_2_·4H_2_O per liter of 0.83 M concentrated hydrochloric acid. Vitamin solution was prepared by dissolving 0.5 g thiamine and 1 mg biotin (per liter of the final medium). Filter sterilized trace metals, MgSO_4_, and vitamin solution were added aseptically to autoclaved medium adjusted to a pH of 7. This prepared BMM has a final Fe concentration of 3.6 µM. To prepare the “no-Fe” medium, FeSO_4_·7H_2_O was omitted from the trace metals. To encourage stronger growth in No-Fe conditions where indicated, MgSO_4_ was added to a concentration of 0.08 mM (BMM8). When required, FeSO_4_·7H_2_O was added to the final concentration of 20 µM, 40 µM, 60 µM, and 100 µM. When needed, 2, 5, or 10 mM CaCl_2_·2H_2_O (Sigma) was added. PAO1 was used to obtain genomic DNA for cloning *efhP*. For DNA manipulations, *E. coli* and *Pa* cultures were grown in Luria–Bertani (LB) broth (per liter: 10 g tryptone, 5 g yeast extract, 5 g NaCl) at 37°C with shaking at 200 rpm. Antibiotics used for *E. coli* were (per mL) 50 µg kanamycin (Kan) or 100 µg carbenicillin (Carb); for *Pa*, (per mL) 60 µg tetracycline (Tet). The plasmid pCTX-1-lux (115) was provided by Dr. Cabeen (Oklahoma State University). *E. coli* SM10 and *Pa* PAO1 were grown in LB broth shaking at 200 rpm for 37°C or on LB agar plates with 1.5% agar at 37°C. For antibiotic selection plates, Tet was used at concentrations of 60 µg/mL (for *Pa*) and 10 µg/mL (for *E. coli*).

**TABLE 1:** Strains, plasmids, and oligonucleotides used for this study

Strain/plasmid	Description	Reference
*P. aeruginosa*		
PAO1	Wild-type strain, Alg−	(116)
PAO1/pMF470	PAO1 with pMF470	This study
Δ*efhP*	Δ*efhP* in PAO1 background	(40)
Δ*efhP*::*efhP*	Δ*efhP* harboring pMF470 plasmid for *efhP* complementation	(40)
ΔPA2657	PAO1 with deletion of *carR*	(117)
PAO1/pREN	PAO1 with pREN	This study
ΔPA2657/pREN	PAO1 with deletion of carR with pREN	This study
13S	R-type pyocin indicator strain	(82)
PAO1	Wild type strain from Imperi lab	(118)
Δ*fur*	PAO1 with deletion of fur and carrying an arabinose-dependent copy of *fur*	(118)
CF1 (102314a)	Clinical isolate from CF patient (age 7)	This study
CF2 (2515D)	Clinical isolate from CF patient (age 17)	This study
CF3 (31314A)	Clinical isolate from CF patient (age 28)	This study
CF4 (22014B)	Clinical isolate from CF patient (age 39)	This study
CF5 (6514C)	Clinical isolate from CF patient (age 55)	This study
*E. coli*		
DH5α	General purpose cloning strain; Δ(lacZ)M15	NEB
SM10	Donor strain used in biparental mating experiments	
ECOREN2	DH5α with pREN	This study
ECOREN3	SM10 with pREN	This study
Plasmids		
pUC19	Cloning vector, Amp^r^	
pMF470	pMF36, 0.52-kb XbaI fragment containing *efhP,* Amp^r^	(40)
pMS402	Promoterless *luxCDABE*	(119)
pREN	pMS402 containing 300 bp efhP promoter region ligated using XhoI and BamHI sites, Tet^r^	This study
Primers		
PA0576_F1	GGGCGAAGAAGGAAATGGTC	(120)
PA0576_R1	CAGGTGGCGTAGGTGGAGAA	(120)
efhP_159_F	AGGCAAGTGTGGTAGCGGCG	This study
efhP_271_R	GATCGGTGTCGGTTCGGGCA	This study
Fur_52_F	CGGGTCAAGATCCTGCAGAT	This study
Fur_219_R	ATCGAAGTTGTGACGCACCA	This study
PA4107_UP_F_XhoI	TAAGCACTCGAGGCCAGGTGGCTGTC	This study
PA4107_DN_R_BamHI	TAAGCAGGATCCGCTTCTTCTCCACAG	This study
16S_533_F	GTGCCAGCAGCCGCGGTAA	(108)
16S_1100_R	AGGGTTGCGCTCGTTG	(108)
prtN_F	GGCAAGTTCGTCGCGGTATC	This study
prtN_R	CCCAGTCCTGAGTGGCGTAG	This study

### Standard DNA procedures

Plasmid DNA was isolated using the Zyppy plasmid miniprep kit (Zymo), and chromosomal DNA was obtained using the Wizard Genomic DNA Purification Kit (Promega). DNA fragments were purified from agarose gels using the Zyppy gel extraction kit. DNA concentration was determined spectrophotometrically using a NanoDrop 1000 Spectrophotometer (Thermo Scientific, Waltham, MA). DNA transformation into *E. coli* DH5α and *Pa* PAO1 were performed using heat shock or electroporation protocols.

### RNA sequencing

For RNA sequencing (RNA-seq) analysis, total RNA was isolated using RNeasy Mini Kit (QIAGEN) from *Pa* PAO1 and ∆*efhP* cultures grown until mid-log phase (12 h) in BMM either supplemented with 5 mM CaCl_2_ or without it to study adaptive response to Ca^2+^. To study rapid response to Ca^2+^, PAO1 and ∆*efhP* cultures were grown until mid-log (12 h) in BMM with no CaCl_2_ and then exposed to 5 mM CaCl_2_ for 10 or 60 min (Fig. S1A). DNase treatment was performed using turbo DNase (Ambion). The absence of genomic DNA was confirmed by conventional PCR using DreamTaq Green PCR Master Mix (Thermo Fisher Scientific) and primers 16s-533-F and 16s-1100-R targeting the 16S rRNA gene. RNA yield was measured using a NanoDrop spectrophotometer (Thermo Fisher Scientific), and the quality of the purified RNA was assessed with an RNA 6000 Nano Chip on a Bioanalyzer 2100 (Agilent) and by 1.2% agarose gel electrophoresis. Following the MIQE guidelines, only the RNA samples with an OD_260_/OD_280_ ratio of 1.8–2.0 and a RIN value of ≥7.0 were selected for further analysis. For sequencing, 250–300 bp insert size, strand-specific libraries were prepared and sequenced in Novogene Co., Ltd. Ribosomal RNA was removed using Ribo-Zero rRNA Depletion Kit (Illumina) and paired-end sequencing (2 × 150 bp) was done on the Nova Seq 6000 platform.

### Analysis of RNA seq reads

All raw RNA sequencing reads were uploaded to KBase (https://www.kbase.us/) for further processing. A minimum of two biological replicates were used for analysis of each strain and condition. Reads were paired and quality-checked using FastQC-v0.11.5 and trimmed with Trimmomatic-v0.36. The trimmed reads were aligned to the genome with HISAT2-v2.1.0 and transcripts were assembled using Cufflinks-v2.2.1. A differential expression matrix was created using DESeq2-v1.20.0 and the resulting raw gene count matrix was uploaded to iDEP.91 (http://bioinformatics.sdstate.edu/idep90/) for further analysis. Principle component analysis and the desired condition comparisons for differential expression as generated on iDEP.91 were utilized for this study (Fig. S1B). The responses of PAO1 and Δ*efhP* to Ca^2+^ were compared in differential expression matrices. The differences in gene expression were presented as log2FC. Significance was determined by adjusted *P* values (*p*_adj) generated by DESeq2. The *p*_adj values below 0.05 were considered statistically significant. Heat maps of selected pathways were generated using Morpheus (https://software.broadinstitute.org/morpheus/). Sorting of the full PAO1 genome into functional categories was made possible using existing annotations from Gene Ontology (http://geneontology.org/), Kyoto Encyclopedia of Genes and Genomes (https://www.genome.jp/kegg/annotation/), and the Pseudomonas Community Annotation Project (PseudoCAP) (https://www.pseudomonas.com/pseudocap), all downloaded in January 2022. Some of the pseudomonas.com gene annotations were updated based on published records. These, for example, included pyocin (121) and ECF (65) encoding genes. All RNA-seq reads utilized for this analysis are accessible in the NCBI Sequence Read Archive database (accession: PRJNA874094).

### Construction of promoter fusion reporter

For studying the transcriptional regulation of *efhP*, a promoterless *luxCDABE* reporter was used. A 300 bp region upstream of *efhP* was amplified from the PAO1 genome by using Phusion polymerase and primers PA4107_UP_F_XhoI and PA4107_DN_R_BamHI (Table 1). The size of the PCR products was verified by agarose gel electrophoresis and purified by using the Zyppy extraction kit (Zymo). The amplified PCR products were digested with BamHI and XhoI and ligated with similarly digested pCTX-1-lux, upstream of the *luxCDABE* genes using the Quick Ligation kit (NEB). For transformation, the ligation mixture was added to heat shock competent DH5α *E. coli* cells and transformed using the protocol described above. The successful transformants were selected on LB agar plates containing 20 µg/mL Tet. The resultant plasmid construct was confirmed by DNA sequencing, named pREN, and was maintained in DH5α, designated as ECOREN2 (Table 1).

### Biparental mating for promoter fusion constructs

The plasmid construct pREN was transformed into the mating strain *E. coli* SM10 by heat shock transformation and designated ECOREN3 (Table 1). It was then conjugated with PAO1 (WT) and the mutant Δ*carR* by biparental mating (122). The donor *E. coli* with the promoter construct and recipient strains were grown in 5 mL of LB overnight at 37°C. The recipient (1 mL) was incubated at 42°C for 2 h. The donor (50 µL) was spot dried on an LB plate in an aseptic zone for an hour. The heat-treated recipient (50 µL) was spot dried on top of the donor and incubated at 37°C for 24 h. The mating spot was scraped and suspended in 1 mL of phosphate-buffered saline (PBS), out of which 10 µL was spread on a Pseudomonas Isolation Agar plate (PIA) supplemented with 60 µg/mL Tet and allowed to grow at 37°C until colonies appeared. Resultant colonies were replica-plated on LB with 60 µg/mL Tet and grown at 37°C until colonies appeared. These colonies were tested for luminescence to verify transformation. Once verified, the strains were grown in LB with 60 µg/mL Tet overnight and stored in 10% skimmed milk at −80°C. Empty pCTX-1-*lux* was transformed into PAO1 to be used as a control using the biparental method as described above.

### Promoter fusion assay

Pa strains carrying pREN were grown in 3 or 5 mL of BMM while shaking at 200 rpm for 12 h. OD_600_ of these cultures was measured and normalized to 0.3 by using sterile BMM. Then it was used to seed a main culture (200 µL final volume of BMM with the desired concentration of Ca^2+^ and/or Fe) at a ratio of 1:100 in 96 well white clear bottom plates (VWR). The plates were covered with lids treated with a mixture of 95% ethanol and 1% Triton solution to prevent condensation. Then the plates were incubated in the Biotek plate reader at 37°C shaking at 200 rpm for 13 h programmed to measure the OD_600_ and luminescence in Relative Light Units (RLU) every 1 h. The resultant RLU values were normalized by OD_600_. Values from non-inoculated cultures were used as blanks. Each experiment was conducted with at least three independent biological replicates, and each experiment was conducted at least twice. Statistical analysis was done by univariate analysis of variance (ANOVA) in SPSS (version 29.0.0.0, IBM Corp. 2022).

### Quantification of pyoverdine production

Intrinsic fluorescence of pyoverdine at 400 nm excitation/460 nm emission (123) was used to quantify pyoverdine production in PAO1, Δ*efhP*, and Δ*efhP*::*efhP* cultures. Colonies grown on LB plates (24 h) containing appropriate antibiotics were used to inoculate precultures in 3 mL BMM8 with no Ca^2+^ and no Fe to deplete intracellular Fe. Following 12 h of growth, the cultures were normalized to an OD_600_ of 0.3, then diluted 1,000× in BMM8 with or without 5 mM Ca^2+^, and 200 µL of the resulting cultures were transferred into black clear-bottom 96 well plates and incubated in BioTek SynergyMx at 37°C with shaking at 200 rpm. Fluorescence (400/460 excitation/emission, 9 nm bandwidth, gain = 50) and OD_600_ were measured hourly. Relative fluorescence units were normalized by OD_600_.

For quantification of pyoverdine production in cultures containing no added Fe, PAO1, ΔefhP, and Δ*efhP*::*efhP* precultures were grown in BMM8 (3.6 µM Fe) to ensure stronger growth. Precultures (12 h) were normalized to an OD_600_ of 0.3 and inoculated (0.1%) into BMM8 No-Fe with or without 5 mM Ca^2+^. Hourly fluorescence and OD_600_ reads were taken in the BioTek SynergyMx as described above. For all pyoverdine assays, at least two independent experiments were conducted, each containing three independent biological replicates. For the cultures of clinical isolates and mutants with different growth patterns, pyoverdine production was calculated as the area under the curve to present total fluorescence produced during the period from early log to after the transition to the stationary phase.

The *fur-*conditional deletion mutant (78) was streaked onto LB plates supplemented with 0.5% arabinose for use in pyoverdine production assay testing the effects of *fur*. Precultures were grown in BMM8 + 0.5% arabinose for 12 h, normalized to an OD_600_ of 0.300, and inoculated (0.1%) into main cultures supplemented with 0 or 5 mM Ca^2+^. Since expression of *fur* in this conditional mutant increases with arabinose induction in a concentration-dependent manner (78), main cultures also included varying concentrations of L-arabinose, ranging from 0 mM to 20 mM. The fluorescence produced was quantified as stated above and normalized by the OD_600_. Three independent biological replicates were averaged to calculate standard error, and at least three independent experiments were performed. Statistical significance was determined by single-factor ANOVA (Microsoft Excel version 16.54) with a cutoff of *P* < 0.05.

### Total Fe quantification by ICP-OES

Samples for ICP-OES were obtained from PAO1 and Δ*efhP* cultures grown at 0 mM and 5 mM Ca^2+^ to mid-log phase (OD_600_ 0.2 ± 0.03) in 100 mL BMM. Following the collection of 15 mL samples from the 0 mM cultures, 5 mM CaCl_2_ (final) was added to the same flasks. Samples were subsequently taken again at 10 min and 60 min Ca^2+^ exposure. The 12 h Ca^2+^ condition utilized cultures grown in the presence of 5 mM Ca^2+^ to mid-log phase. All samples were spun at 6,010 *× g* for 10 min at 4°C, then supernatants were passed through 0.2 µm filters. Filtered supernatants were stored at −20°C until ICP-OES analysis. ICP-OES was performed using the iCAP PRO XPS ICP-OES Duo Spectrometer coupled with Qtegra ISDS software. A wavelength of 259.940 nm (Aqueous-Radial-iFR) was selected to detect Fe specifically, limiting interference, in samples. Measurements performed in parts per million were converted to μM for analysis. Three independent biological replicates were averaged, and standard error was calculated. Significance was determined by single-factor ANOVA (Microsoft Excel version 16.54) with a cutoff of *P* < 0.05.

### Pyoverdine spectral measurements

PAO1 was grown for 16 h in 5 mL BMM8 (0 µM Fe, 0 mM Ca^2+^). Cultures were spun at 9,319 × *g* for 10 min and filter sterilized. Absorbance spectral scans of 1 mL filtrate samples were conducted in cuvettes in a BioTek SynergyMx. CaCl_2_.2H_2_O dissolved in H_2_O was added to a concentration of 5 mM in appropriate samples. Equal volumes of FeCl_3_·6H_2_O dissolved in deionized H_2_O were added to filtrates at concentrations of 4 µM, 20 µM, 60 µM, and 1 mM FeCl_3_ following the addition of either CaCl_2_ or H_2_O control. Deionized H_2_O without FeCl_3_ was added to 0 µM Fe samples. All samples were incubated for 30 min at room temp before absorbance (Abs) reads. Raw Abs values were normalized by BMM8 blank. Percentage-bound pyoverdine was calculated using the pH-insensitive specific pyoverdine-Fe Abs460 shoulder (124). Abs460 values for each sample were divided by Abs460 of Fe-saturated pyoverdine (1 mM FeCl_3_, 0 mM Ca^2+^). Three technical replicates were conducted. Significance was determined by single-factor ANOVA (Microsoft Excel version 16.54) with a cutoff of *P* < 0.05.

### *efhP* transcription during interactions with epithelial cells

Two types of human lung epithelial cell lines were used: adenocarcinoma alveolar basal epithelial cells A549 (ATCC CCL-185) (125) and CF bronchial CuFi-5 (ATCC CRL-4016) epithelial cells which are homozygous for the ∆F508 mutation of the CFTR protein in CF (126). A549 cell line in RPMI medium (ThermoFisher, MA) or CuFi-5 cell line in Airway Epithelial Cell Growth Medium (AECGM, PromoCell, Heidelberg, Germany) were seeded into six-well tissue culture plates and incubated at 37°C in 5% CO_2_ until reaching 100% confluence controlled by phase-contrast microscopy. PAO1 was grown in BMM until mid-log phase (12 h; OD_600_ = 0.18 ± 0.03). The bacterial cells were harvested, resuspended in the appropriate cell culture medium, and inoculated to the apical cell surface at a multiplicity of infection of 30. Cells were incubated for 2 h, extracellular bacteria were removed, media containing 200 µg/mL gentamicin (Gm) was added, and cells were incubated for an additional 4 h. For sampling of the invaded PAO1, cells were washed with PBS and lysed in 1 mL of ultrapure water. For sampling of the adhered bacteria, cells were incubated in the absence of Gm for 6 h, washed with PBS, and harvested. This bacterial population also contained invaded bacteria. To count the bacterial cell number, the samples were serially diluted 10×, spotted onto LB agar plates, and colony forming units (CFU) were calculated. For RT-qPCR, total RNA was extracted as described above, and cDNA was synthesized using a Transcriptor first-strand cDNA synthesis kit (Roche) and RT-qPCR was performed using SYBR green master mix (Roche, Indianapolis, IN) and a Light Cycler 480 Instrument II (Roche) as described previously (127). The sequences of PCR primers used for RT-qPCR are provided in Table 1. The *rpoD* gene was used as an internal control. The relative fold changes in gene expression levels were calculated using the 2^−ΔΔCt^ method (128, 129).

### *efhP* transcription in CF clinical isolates

The clinical isolates from CF patients (age 7 to 55 years) were isolated from sputum samples collected from patients at the Children’s Hospital CF clinic in Oklahoma City, Oklahoma. Sputum samples were streaked on PIA and incubated at 37°C overnight until individual colonies appeared. Isolates were grown in liquid LB medium for 16 h and frozen stocks made in 10% skim milk before storage at −80°C. To quantify *efhP* transcription via RT-qPCR, the strains were grown in LB medium until the mid-log phase (OD_600_ of 0.30 ± 0.05). LB was used as the isolates grew poorly in BMM8. Total RNA was isolated, and cDNA was synthesized as described in reference 35. The absence of genomic DNA (gDNA) in the RNA samples was verified by PCR amplification of 16S *rRNA* genes prior to cDNA synthesis by using DreamTaq green PCR master mix, alongside a gDNA positive control. If gDNA was present, samples were run through a second DNase treatment.

RT-qPCR was performed using SYBR green master mix (Roche, Indianapolis, IN) and a Light Cycler 480 Instrument II (Roche). Five serial dilutions of gDNA (10^1^–10^−3^ ng) were run as templates to plot a standard curve for *efhP* and *rpoD* primers. In a base-10 semi-logarithmic graph, the threshold cycle (Cp) values were plotted against the dilution factor, and an exponential trendline was fitted to the graph. Only a correlation coefficient (*R*^2^) greater than 0.99 was accepted for data analysis. The trendline equation was then used to determine the relative expression levels of amplified genes. Expression of the *rpoD* gene was used as a reference, and the relative *efhP* expression was calculated as the ratio *efhP/rpoD*. All primers used are listed in Table 1. Statistical significance was determined by single-factor ANOVA (Microsoft Excel version 16.54) with a cutoff of *P* < 0.05.

### Validation of *fur* and *prtN* transcriptional regulation

Wild-type PAO1 was grown alongside Δ*efhP* in BMM with or without 5 mM CaCl_2_ to mid-log phase. Cells were harvested upon reaching an OD_600_ of 0.2 ± 0.05 (~12 h). RNA was then isolated as described above, and cDNA was synthesized using a Transcriptor first-strand cDNA synthesis kit (Roche). Primers for *fur* and *prtN* (Table 1) were tested by qPCR to ensure amplification efficiency in relation to internal control *rpoD* or *nadB*. RT-qPCR was then performed using SYBR green master mix (Roche, Indianapolis, IN) and a Light Cycler 480 Instrument II (Roche) as described previously (127). The relative fold change in *fur* expression was calculated using the 2^−ΔΔCt^ or standard curve methods (128, 129). Statistical significance was determined by single-factor ANOVA (Microsoft Excel version 16.54) with a cutoff of *P* < 0.05.

### Pyocin assay

Wild-type PAO1, Δ*efhP*, and Δ*efhP::efhP* were grown in BMM8 with or without 5 mM CaCl_2_ to mid-exponential phase. Cultures were normalized to OD_600_ 0.3, pelleted, and filter sterilized. In parallel, overnight cultures of the R-type pyocin indicator strain, 13S, were normalized to OD_600_ 0.3. The supernatants of PAO1, Δ*efhP*, or Δ*efhP::efhP* (100 µL) were added to 100 µL of the normalized pyocin-indicator strain in 1.5 mL centrifuge tubes. The tubes were incubated for 30 min at 37°C. CFUs were determined following serial 10× dilution and plating on LB plates. CFUs from before and after treatment were used to calculate percent survival. To determine the difference in survival as a Ca^2+^ effect, the fold change of percent survival was calculated by dividing percent survival at 5 mM Ca^2+^ by that at 0 mM Ca^2+^ condition. Treatments were performed in triplicates, the percent survival was averaged and used to calculate the fold difference between different Ca^2+^ levels. Each experiment was repeated independently three times.

## Data Availability

All raw RNA sequencing reads were uploaded to KBase (https://www.kbase.us/). All RNA-seq reads utilized for this analysis are accessible in the NCBI Sequence Read Archive database (accession: PRJNA874094).

## SUPPLEMENTAL MATERIAL

Supplemental material mbio.02447-24-s0001.pdf Fig. S1 to S3 and Table S1.

ASM does not own the copyrights to Supplemental Material that may be linked to, or accessed through, an article. The authors have granted ASM a non-exclusive, world-wide license to publish the Supplemental Material files. Please contact the corresponding author directly for reuse.
