# Supplementary material for: EF-hand calcium sensor, EfhP, controls transcriptional regulation of iron uptake by calcium in Pseudomonas aeruginosa
Source: mBio. 2024 Oct 22;15(11):e02447-24. doi: 10.1128/mbio.02447-24 (PMC11559002; doi:10.1128/mbio.02447-24)
Supplement: Supplemental material — Fig. S1 to S3 and Table S1. [file mbio.02447-24-s0001.pdf]

A

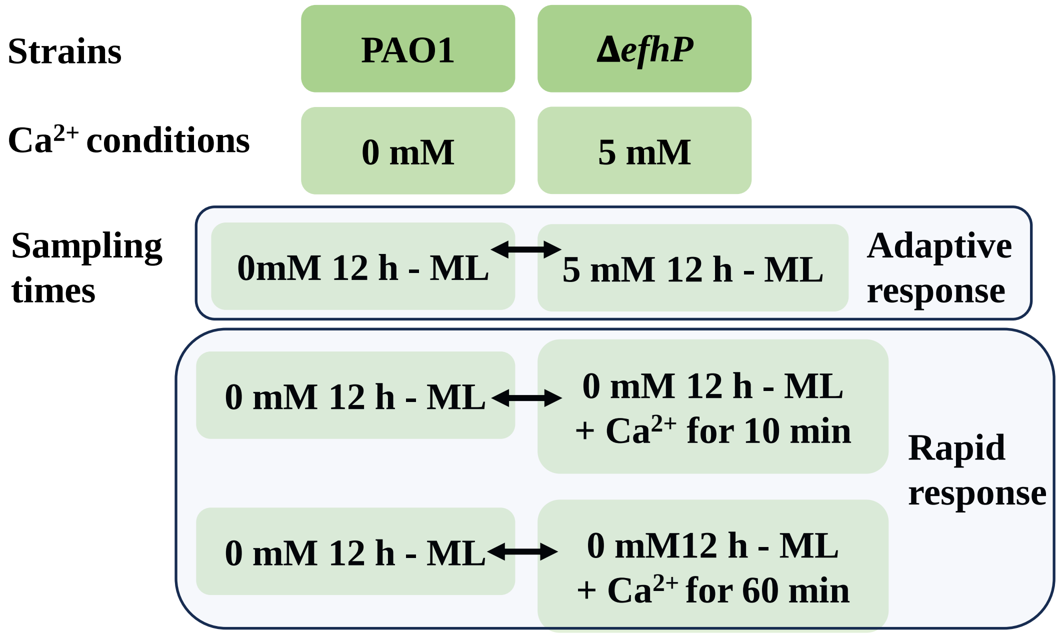

B

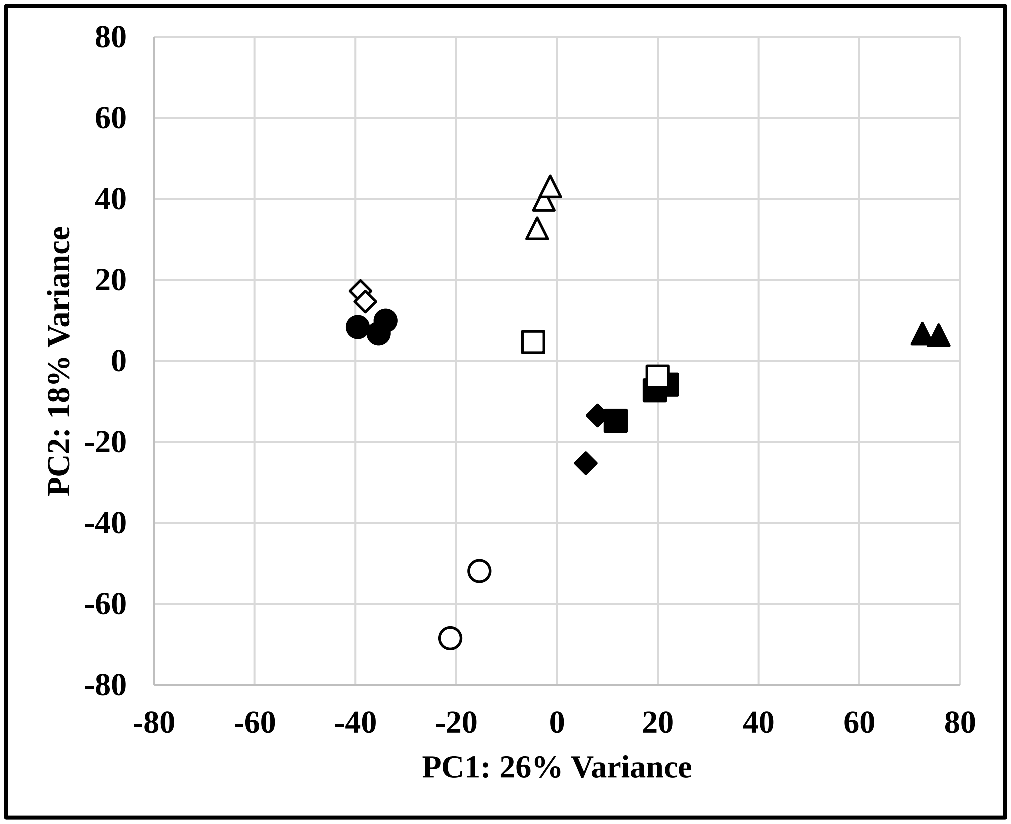

Figure S1. RNA sequence design and analysis. (A) Schematic showing RNA-seq experimental design. This study utilized two *Pa* strains, two Ca^{2+} concentrations, and three sampling times, for a total of 12 unique conditions. (**B**) Principal component analysis (PCA) plot of RNA sequencing reads selected for further analysis. RNA seq samples for PAO1 (black) and **Δ***efhP* (white). Ca^{2+} exposures of 0 mM (circle), 10 min (triangle), 60 min (diamond), and 12 h (square). PCA was generated on iDEP.91 for this study.

A

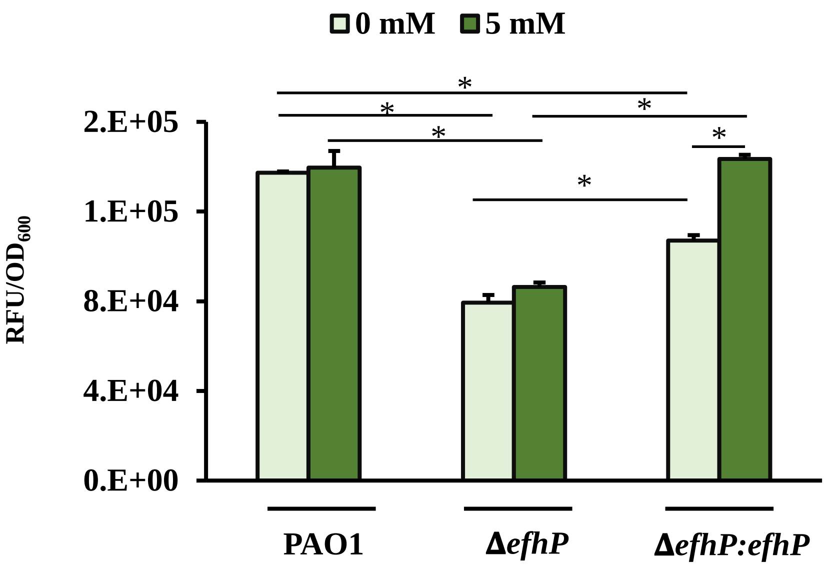

B

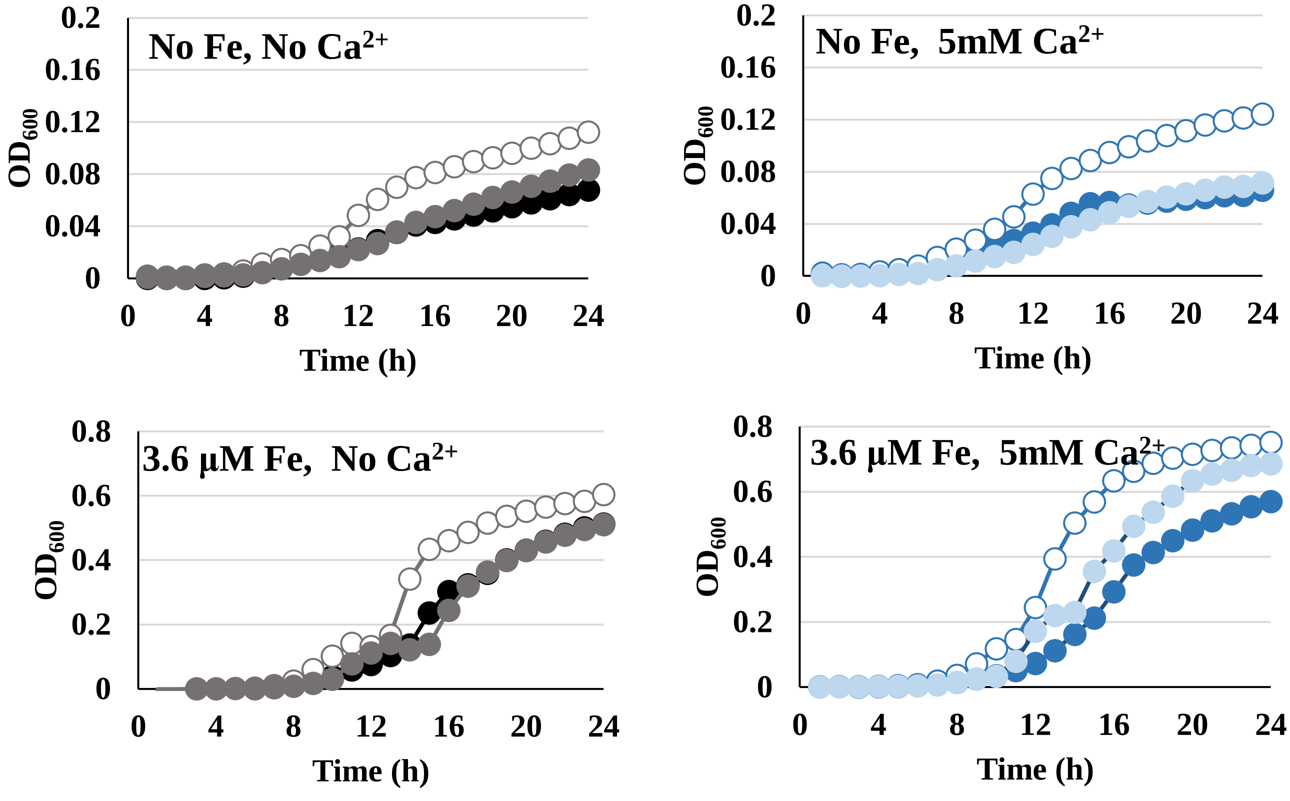

Figure S2 Growth and pyoverdine production of *Pa* at no added Fe. (A) Stationary phase (24 h) pyoverdine production of no Fe BMM8 cultures of strains PAO1, **Δ***efhP*, and **Δ***efhP:efhP* was quantified by fluorescence at 400 excitation/460 emission and normalized by OD_{600}. Statistical significance determined by single factor ANOVA (Microsoft Excel v16.54) with a p of < 0.05 (*). (**B**) Growth of PAO1 (dark), **Δ***efhP* (white), and **Δ***efhP:efhP* (light) in no-Fe BMM8 in the absence or presence of 5 mM Ca^{2+} and 3.6 µM Fe. Data represents hourly averaged OD_{600} values of three independent biological replicates. The standard error bars were plotted but are not visible due to their small values.

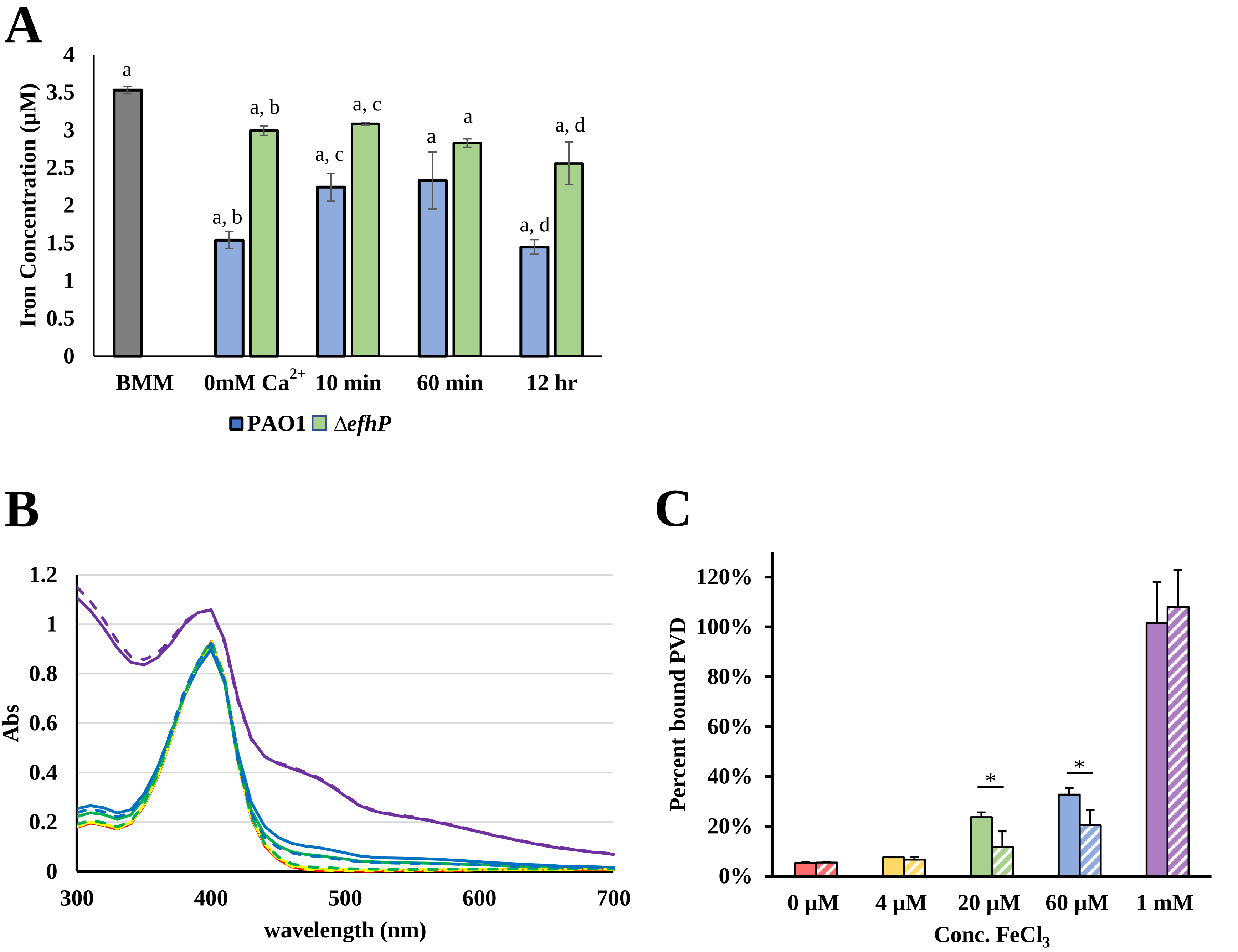

Figure S3. Total iron quantification of mid-log cultures (A) and spectral measurements of PVD-Fe complex formation (B-C). (A) ICP-OES was employed to quantify total iron remaining in pelleted cultures of PAO1 and **Δ*efhP*** grown under same conditions as used for RNA sequencing. Data was converted from parts per million to µM for analysis. Statistical significance was determined by single factor ANOVA (* indicates $p < 0.05$): a, significant against BMM control medium; b, significant between PAO1 and Δ*efhP* at 0 mM Ca^{2+} condition; c, significant between PAO1 and Δ*efhP* at 10 min Ca^{2+} condition; d, significant between PAO1 and Δ*efhP* at 12 hr Ca^{2+} condition. **(B)** Absorbance spectral scan of pyoverdine-rich filtrate in No-Fe BMM8 containing 0 µM (red), 4 µM (yellow), 20 µM (green), 60 µM (blue), or 1 mM (purple) added $FeCl_3$. Scans were conducted in the presence (dashed) or absence (solid) of 5 mM $CaCl_2$. **(C)** Percentage of bound pyoverdine following addition of each indicated $FeCl_3$ concentration in the presence (dashed) or absence (solid) of 5 mM $CaCl_2$. Abs_{460} of each sample was divided by the average Abs_{460} of the saturated 1 mM $FeCl_3$ 0 mM $CaCl_2$ samples to calculate percentages bound. Significance determined by single factor ANOVA ($p < 0.05$ = *)

Table S1. CFU results for PAO1 populations recovered after infecting A549 or CuFi-5 epithelial cells. Control condition reflects PAO1 grown in cell culture media described in Methods and in the absence of epithelial cells.

Epithelial cell line	PAO1	CFU (/ml)
A549	Control	1.27E+08 ± 7.7E+06
	Planktonic	1.33E+08 ± 1.3E+07
	Adhered + Invaded	2.30E+06 ± 5.8E+05
	Invaded	5.30E+04 ± 1.3E+05
CuFi-5	Control	2.70E+08 ± 2.2E+07
	Planktonic	2.57E+08 ± 2.2E+07
	Adhered + Invaded	2.20E+07 ± 4.1E+07
	Invaded	9.00E+04 ± 3.7E+04
